# Empagliflozin prevents oxidative stress in human coronary artery endothelial cells via the NHE/PKC/NOX axis

**DOI:** 10.1016/j.redox.2023.102979

**Published:** 2023-12-02

**Authors:** Xiaoling Li, Mengnan Wang, Jan-Ole Kalina, Benedikt Preckel, Markus W. Hollmann, Martin Albrecht, Coert J. Zuurbier, Nina C. Weber

**Affiliations:** a Amsterdam, University Medical Centers, Location AMC, Department of Anesthesiology, Laboratory of Experimental Intensive Care and Anesthesiology-L.E.I.C.A, Amsterdam Cardiovascular Science (ACS), Meibergdreef 11, 1105 AZ, Amsterdam, the Netherlands; b Department of Anesthesiology and Intensive Care Medicine, Universitätsklinikum Schleswig-Holstein, Campus Kiel, 24105, Kiel, Germany

**Keywords:** Sodium glucose co-transporter 2 inhibitor (SGLT2i), Cyclic stretch, Human coronary artery endothelial cells (HCAECs), Nicotinamide adenine dinucleotide phosphate oxidase (NOX), Reactive oxygen species (ROS), Intracellular calcium (Ca^2+^)

## Abstract

**Background:**

Empagliflozin (EMPA) ameliorates reactive oxygen species (ROS) generation in human endothelial cells (ECs) exposed to 10 % stretch, but the underlying mechanisms are still unclear. Pathological stretch is supposed to stimulate protein kinase C (PKC) by increasing intracellular calcium (Ca^2+^), therefore activating nicotinamide adenine dinucleotide phosphate oxidase (NOX) and promoting ROS production in human ECs. We hypothesized that EMPA inhibits stretch-induced NOX activation and ROS generation through preventing PKC activation.

**Methods:**

Human coronary artery endothelial cells (HCAECs) were pre-incubated for 2 h before exposure to cyclic stretch (5 % or 10 %) with either vehicle, EMPA or the PKC inhibitor LY-333531 or PKC siRNA. PKC activity, NOX activity and ROS production were detected after 24 h. Furthermore, the Ca^2+^ chelator BAPTA-AM, NCX inhibitor ORM-10962 or NCX siRNA, sodium/potassium pump inhibitor ouabain and sodium hydrogen exchanger (NHE) inhibitor cariporide were applied to explore the involvement of the NHE/Na^+^/NCX/Ca^2+^ in the ROS inhibitory capacity of EMPA.

**Results:**

Compared to 5 % stretch, 10 % significantly increased PKC activity, which was reduced by EMPA and PKC inhibitor LY-333531. EMPA and LY-333531 showed a similar inhibitory capacity on NOX activity and ROS generation induced by 10 % stretch, which was not augmented by combined treatment with both drugs. PKC-β knockdown inhibits the NOX activation induced by Ca^2+^ and 10 % stretch. BAPTA, pharmacologic or genetic NCX inhibition and cariporide reduced Ca^2+^ in static HCAECs and prevented the activation of PKC and NOX in 10%-stretched cells. Ouabain increased ROS generation in cells exposed to 5 % stretch.

**Conclusion:**

EMPA reduced NOX activity via attenuation of the NHE/Na^+^/NCX/Ca^2+^/PKC axis, leading to less ROS generation in HCAECs exposed to 10 % stretch.

## Highlights

• EMPA prevents stretch induced ROS production in endothelial cells by inhibiting PKC (85 characters)
• The PKC inhibition by EMPA in stretched cells is mediated by Ca^2+^ reduction (77 characters)
• EMPA lowers intracellular Ca^2+^ in endothelial cells through the NHE/Na^+^/NCX axis (82 characters)

## Graphical abstract

**Figure:**
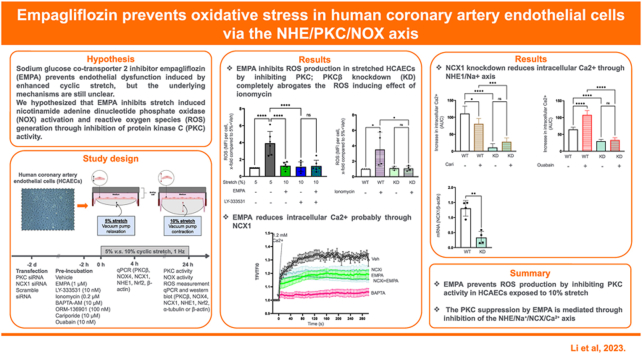

## Abbreviations

AUC: Area under the curve
Ca^2+^: Calcium
ECs: Endothelial cells
EMPA: Empagliflozin
HBSS: Hank's Balanced Salt Solution
HCAECs: Human coronary artery endothelial cells
MFI: Mean fluorescence intensity
Na^+^: Sodium
NCX: Sodium calcium exchanger
NHE: Sodium hydrogen exchanger
NO: Nitric oxide
NOX: Nicotinamide adenine dinucleotide phosphate oxidase
PKC: Protein kinase C
PMA: Phorbol 12-myristate 13-acetate
ROS: Reactive oxygen species
SGLT2i’s: Sodium glucose co-transporter 2 inhibitors
TFI: Total fluorescence intensity
TNF-α: Tumor necrosis factor-α
VE-cadherin: Vascular endothelial-cadherin

## 1 Introduction

The sodium glucose co-transporter 2 inhibitor (SGLT2i) empagliflozin (EMPA) has been approved for the therapy of heart failure given the cardiovascular benefits in patients with and without diabetes mellitus [1,2]. Cardiovascular protection of EMPA may be partially explained by its direct effect on endothelial cells (ECs) [3].

Under physiological conditions, ECs form a cell monolayer that maintain haemostasis of the cardiovascular system by producing endothelium-derived vasoactive factors, preventing monocyte adhesion and transmigration, as well as regulating the contraction and relaxation of cardiomyocytes [4]. Upon exposure to pathological stimuli (e.g. enhanced cyclic stretch, hyperglycaemia), ECs undergo multiple dysregulations like excessive reactive oxygen species (ROS) production, an excessive inflammatory response and increased cell permeability, thereby contributing to the development of cardiovascular disease [5]. Excessive ROS production plays a crucial role in endothelial dysfunction: ROS activate Src family kinase to phosphorylate vascular endothelial (VE)-cadherin, leading to VE-cadherin loss, adherens junction disruption and increase in cell permeability [6].

EMPA demonstrates a potent anti-oxidative effect in ECs. Live cell image demonstrated that EMPA suppresses ROS production and restores NO bioavailability in static human ECs stimulated with tumor necrosis factor-α (TNF-α) [7,8]. Recently, we showed for the first time that SGLT2i’s also inhibit increased ROS production and cell permeability in human coronary artery endothelial cells (HCAECs) exposed to 10 % stretch, revealing that EMPA also exerts protective effects in dynamic cultured ECs [9]. The nicotinamide adenine dinucleotide phosphate oxidases (NOX)1/4 inhibitor GKT136901 lowers ROS in ECs undergoing 10 % stretch to a similar extent as EMPA does, and its ROS reducing capacity is not amplified when combined with EMPA, supporting the involvement of NOX as a key mediator in the anti-oxidative effect of EMPA [9].

Exposure to TNF-α and cyclic stretch increases activity of sodium-hydrogen exchanger (NHE) in ECs and cardiomyocytes, leading to the increase in intracellular sodium (Na^+^) [10,11]. It is speculated that Na ^+^ triggers ROS generation inside ECs by enhancing cytosolic Ca^2+^ through the sodium calcium exchanger (NCX), consequently stimulating Ca^2+^ dependent protein kinase C (PKC) isoforms [3,10,12]. Elevated PKC activity, especially PKC-β, has a dominant role in NOX activation and ROS production in human ECs [13]. Previous studies showed that SGLT2i’s directly inhibit NHE [10,[14], [15], [16]], and that lowering NHE activity with EMPA or cariporide suppressed ROS production in human ECs subjected to enhanced stretch and TNF-α [9,10]. Yet, the effect of EMPA on intracellular Ca^2+^ and PKC activity was not investigated in the these studies.

We hypothesized that EMPA inhibits stretch-induced NOX activation and ROS generation through preventing PKC activation. We aimed to explore the involvement of PKC in the ROS reducing effect of EMPA in stretched ECs, as well as the upstream signalling pathway for PKC inhibition by EMPA.

## 2 Methods

### 2.1 Cell culture and cyclic stretch

HCAECs were purchased from commercial supplier (PromoCell, Heidelberg, Germany) and maintained in endothelial cell basal medium MV2 (PromoCell) containing 10 % fetal bovine serum (FBS, TICO, Antwerpen, Belgium) and recommended supplements (PromoCell). To reduce individual variation, cells from different donors were pooled together. Cells were seeded onto BioFlex® 6-well plates (Flexcell International, McKeesport, PA, USA) as described previously and all experiments were performed at passage 5 to 7 [9]. HCAECs were pre-incubated with either vehicle or 1 μM EMPA (MedChemExpress, New York, NJ, USA) for 2 h before exposure to 4 or 24 h cyclic stretch (1 Hz) using the FX-6000T^TM^ Tension System. Based on previous *in vitro* studies, 5 % stretch was considered as physiological control and 10 % as damage model [9,17]. During cyclic stretch, the FBS concentration inside ECBM MV2 was reduced to 2 % to prevent the binding of EMPA to serum proteins (over 80 % in vivo) [18]. 18 h starvation in ECBM with 2 % FBS was applied in the current study in order to minimize the effect of serum reduction on the metabolism of HCAECs [19,20].

To investigate the underlying mechanisms of the anti-oxidative effect of EMPA in 10%-stretched cells, 10 nM LY-333531 (Abcam, Cambridge, UK) was used to inhibit PKC activity, 10 nM phorbol 12-myristate 13-acetate (PMA, Sigma-Aldrich, St. Louis, MO, USA) to activate PKC, 5 μM BAPTA-AM (Abcam; or Thermo Fisher, Waltham, MA, USA) to chelate intracellular Ca^2+^, 0.2 μM Ionomycin (Sigma-Aldrich) to increase cytosolic Ca^2+^, 100 nM ORM-10962 (MedChemExpress) to inhibit NCX activity, 10 μM cariporide (MedChemExpress) to block NHE activity, and 10 or 100 nM ouabain (Thermo Fisher) to inhibit the sodium-potassium pump and increase cytosolic Na^+^. Additionally, knockdown of PKCβ and NCX1 were performed to evaluate the participation of these specific genes in the anti-oxidative effect of EMPA.

### 2.2 Cell lysis and PKC activity measurement

After 24 h, whole cell lysates were collected for further experiments. Briefly, cells were washed twice with ice cold PBS and collected in homogenisation buffer (0.25 M sucrose and 0.02 M HEPES) with 0.5 % triton X-100, 1 mM dithiothreitol and 1 % Halt^TM^ Protease and Phosphatase Inhibitor Single-Use Cocktail (Thermo Fisher). Samples were sonicated on ice with repeated cycles, followed by 10 min centrifugation with 12000 g at 4 °C. Supernatants were collected and stored at −80 °C until further use.

PKC activity was measured from cell lysates with a PKC Kinase Activity Assay Kit (Abcam, ab139437), following the instruction provides by the manufacturer.

### 2.3 NOX activity measurement

Activity of NOX was detected from cell lysates using the protocol modified from Vincent et al. [21]. Briefly, samples were diluted to 1:2 ratio with de-ionized water and loaded onto a 96-well clear flat-bottom plate (50 μl/well) together with 50 mM Tris-MES buffer (100 μl/well) and 600 μM nicotinamide adenine dinucleotide hydrogen (NADH, 50 μl/well). The change in absorbance of NADH at 340 nm (optical density, OD) was recorded for 30 min at 37 °C with the SpectraMax M2e Microplate Reader (Molecule Devices, San Jose, CA, USA). NOX activity is determined by the reduction rate of NADH absorbance (OD/min), and all the results were adjusted to the protein amount per well.

Cell lysates from static HCAECs receiving 50 μM pyocaynin were used as positive control, and 1 μM NOX1/4 inhibitor GKT136901 was added to samples during measurements to test the specificity of NOX activity [9].

### 2.4 ROS measurement

After 24 h stretch, 5 μM CellROX® Deep Red Reagent (Thermo Fisher) was applied to the cells for 30 min to measure ROS, following the previously reported protocol [9]. Cells were then washed once with pre-warmed PBS and fixed for 15 min with 3.7 % formaldehyde. Then, the silicone membranes with cells were loaded on glass coverslips with mounting fluid containing DAPI (Thermo Fisher) and imaged with a Leica DM6 Wide-Field Microscope (Leica Microsystems) at 400 × magnification. At least 6 cell areas were imaged randomly from each well and a total fluorescence intensity (TFI) for the cells in every area were measured using image J1.8.0. The TFI was divided by the cell number to generate a mean fluorescence intensity (MFI) for each cell. Within each experiment, all data were normalized to the control group. To reduce bias, ROS quantification was carried out by two independent blinded investigators (X.L. & J.O.K. or M.W.).

### 2.5 Cell permeability assay

Cell permeability was visualized as described previously [9,17]. Briefly, BioFlex plates were pre-coated overnight with 0.25 mg/ml biotinylated gelatin (3 ml/well) and washed with PBS before seeding. After 24 h stretch, 25 μg/ml fluorescein isothiocyanate labelled avidin was applied on the HCAECs monolayer for 3 min, followed by three times washing steps to remove unbound probe. The membranes were then loaded onto glass coverslips with mounting fluid containing DAPI (Thermo Fisher) and imaged using Leica DMi8 Advanced Light Microscopy (Leica Microsystems) at 200 × magnification. images from each treatment were acquired randomly, and the cell permeability was quantified by MFI per cell in J1.8.0 in a blinded manner (X.L. & J.O.K.).

### 2.6 Infra-red western blot

Total protein concentrations in cell lysates were determined by the Lowry method and adjusted to 1000 or 2000 μg/ml before loading on the gel. Infra-red western blot was performed as described before [9,22]. Membranes were washed three times after overnight incubation with primary antibodies (NHE1, 1:500, Abcam; NCX1, 1:500, Abcam; NOX4, 1:500, Thermo Fisher; PKCβ, 1:500, Proteintech, Rosemont, IL, USA; VE-cadherin, 1:1000, CST; SOD-1, 1:5000, Abcam; α-tubulin, 1:40000 or 1:20000, Thermo Fisher), followed by 1 h incubation with the complementary secondary antibodies (1:5000, Li-Cor, Lincoln, USA). Then, membranes were scanned with the Odyssey CLx operator (Li-Cor) and quantifications of the signals of each band was performed with Image Studio^TM^ Software (Version 5.2, Li-Cor).

### 2.7 Immunofluorescence staining

After 24 h stretch, HCAECs were fixed for 10 min with 3.7 % formaldehyde, followed by three times washing with PBS and 30 min blocking with 1 % bovine serum albumin. Cells were then incubated for 1 h at room temperature with an anti-VE-cadherin monoclonal antibody (1:60, eBioscience, San Diego, CA, USA) and unbound antibody was washed away with PBS. Afterwards, a secondary antibody (1:400, Thermofisher) was applied on cells for 30 min. Cells were covered with mounting fluid containing DAPI (Thermofisher). VE-cadherin was imaged using Leica TCS SP8 X Confocal Microscope (Leica microsystems) at 630 × magnification.

### 2.8 Calcium measurement

Briefly, HCAECs grown in 12-well plates were incubated for 30 min with 2 μM Fluo4-AM, which was diluted in Ca^2+^ free Hank's Balanced Salt Solution (HBSS, Gibco, Waltham, MA, US) containing vehicle, 1 μM EMPA, 100 nM ORM-10962, 5 μM BAPTA-AM or 10 μM cariporide. Cells were then subjected to 30 min de-esterification in probe free HBSS with the respective treatment after one time washing with HBSS. 100 nM Ouabain was applied on cells during de-esterification to investigate the causal relationship between an increase in intracellular Na^+^ and intracellular Ca^2+^ in HCAECs..0.2 μM Ionomycin was added onto HCAECs to enhance intracellular Ca^2+^.

Cells were imaged for five times every 5 s to obtain a baseline value of the total fluorescence intensity (TFI0). Thereafter, 1.2 mM Ca^2+^ was loaded on the cells and the change of fluorescence intensity was recorded for 5 min every 5 s. Change of intracellular Ca^2+^ was indicated by the ratio of measured TFI to TFI0 (TFI/TFI0), and increase in Ca^2+^ was quantified using the area under curve (AUC) after application of 1.2 mM Ca^2+^.

### 2.9 Transfection with small interfering RNA (siRNA)

The knockdown of NCX1 and PKC-β was achieved using 10 nM NCX Human siRNA Oligo Duplex (OriGene, Rockville, MD, USA) and 25 nM PKC-β Human siRNA Oligo Duplex (OriGene), respectively. 10 nM or 25 nM scrambled siRNA (OriGene) was used as negative control.

HCAECs were seeded into 6-well plates at a density of 50000 cells/well to reach a confluence of 50–80 % after 2 days. Before transfections, cells were starved with medium containing 2 % FBS for 24 h siRNA Oligo Duplexes or scrambled siRNA were mixed together with Lipofectamine RNAiMAX (Thermo Fisher) and incubated for 20 min. The mixture was added onto cells and incubated for 24 h with antibiotic-free ECBM MV2 containing 10 % FBS. Transfected cells were then split into BioFlex® 6-well plates or 12-well plates for subsequent experiments.

### 2.10 Total RNA extraction and quantitative polymerase chain reaction (qPCR)

After 4 or 24 h stretch, cells were lysed with TRIzol™ Reagent (Invitrogen) and total RNA was extracted from cell lysates following the procedures suggested by the manufacturer (Pub. No. MAN0001271, Invitrogen). The concentration and purity of total RNA was measured using NanoDrop 2000/2000c spectrophotometer (Thermofisher). 1 μg total RNA was reverse transcribed into cDNA after 10 min incubation with Oligo(dT) Primer (Thermo Fisher) at 65 °C, followed by 5 min incubation with Reverse Transcriptase and dNTP mix (Thermo Fisher) at 85 °C.

Afterwards, cDNA solutions were mixed with SYBR™ Green PCR Master Mix (Thermo Fisher) and primers for target genes (NCX1, NHE1, NOX, Nrf2, PKC-β and β-actin). Quantification of mRNA expression was performed using the LinRegPCR system (version 11.0). Sequences of primers are shown in Table S1.

### 2.11 Sample size calculation and statistical analysis

Five independent experiments in each group are required to detect a 25 % difference between the vehicle and the intervention group, given a standard deviation of 10 %, a power of 80 % and an α of 0.05 [8,9,22].

Statistical analyses were performed using GraphPad Prism 8.3.0 (GraphPad, San Diego, CA, USA). All data were checked for normal distribution using Shapiro-Wilk test and are presented as mean ± standard deviation (SD). Student t-test or one-way ANOVA with Bonferroni post hoc correction was used when comparing the differences between groups. *P < 0.05*, referred as *; ** for *P < 0.01*, *** for *P < 0.001* and **** for *P < 0.0001*, were considered statistically significant.

## 3 Results

### 3.1 EMPA ameliorates stretch induced endothelial dysfunction via inhibiting PKC activity

**Fig. 1:**
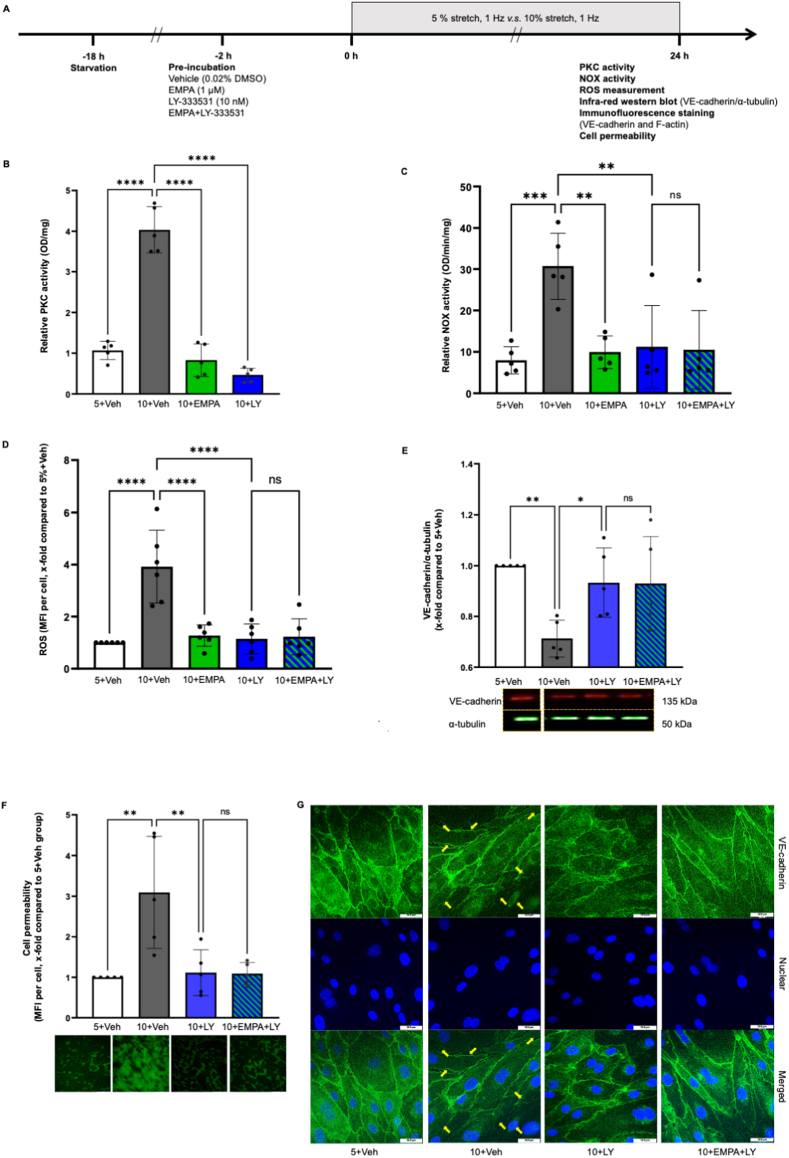
**EMPA attenuates stretch induced oxidative stress and cell permeability via inhibiting PKC activity.** Cells were pre-incubated for 2 h with vehicle, 1 μM EMPA, 10 nM LY-333531, or EMPA plus LY-333531, before exposure to 5 % stretch or 10 % stretch for 24 h, and the flow chart is shown in Fig. 1A. PKC and NOX activity was measured from cell lysates (B & C, n = 5). ROS and cell permeability were quantified using mean fluorescence intensity (MFI) and all the data were normalized to 5+Veh group in D and F (D, n = 6; F, n = 5). Expression and integrity of VE-cadherin was detected with infra-red western blot (E, n = 5) and immunofluorescence staining (G, n = 3), respectively. Representative images and blots are shown in the right/lower panel and data are presented as mean ± SD. *P < 0.05*, referred as *; ** for *P < 0.01*, *** for *P < 0.001* and **** for *P < 0.0001*, were considered statistically significant. Non-normalized ROS values of each experiment are presented in Fig. S9 and Table S2. (For interpretation of the references to colour in this figure legend, the reader is referred to the Web version of this article.)

Compared to 5 % stretch, 10 % stretch increased PKC activity in HCAECs (Fig. 1B, OD/mg, 5+Veh: 1.07 ± 0.23 *vs* 10+Veh: 4.03 ± 0.57, *P < 0.05*), which was completely reverted by both 1 μM EMPA and 10 nM LY-333531 (Figs. 1B and 10+EMPA: 0.83 ± 0.40, 10+LY: 0.47 ± 0.17, *P* both*<0.05 vs* 10+Veh). EMPA and LY-333531 both prevented NOX activity in HCAECs subjected to 10 % stretch (Fig. 1C, OD/min/mg, 5+Veh: 7.96 ± 3.29 *vs* 10+Veh: 30.70 ± 8.02, *P < 0.05*; 10+EMPA: 9.93 ± 3.94, 10+LY: 11.20 ± 9.98, *P* both*<0.05 vs* 10+Veh), which was not augmented by combined treatment of both drugs (Figs. 1C and 10+EMPA + LY: 10.52 ± 9.46, *P > 0.05 vs* 10+LY). These data indicated that EMPA inhibits NOX activation by suppressing PKC activity in 10%-stretched HCAECs.

EMPA and LY-333531 both lowered the 10 % stretch induced ROS generation, and a combined treatment exerted no additional effect (Fig. 1D, x-fold compared with 5+Veh, 10+EMPA: 1.25 ± 0.42, 10+LY: 1.14 ± 0.58, *P* both*<0.05 vs* 10+Veh: 3.90 ± 1.40; 10+EMPA + LY: 1.22 ± 0.70, *P > 0.05 vs* 10+LY). LY-333531 restored the VE-cadherin loss and cell permeability increase in cells subjected to 10 % stretch (Fig. 1E, VE-cadherin/α-tubulin, x-fold compared with 5+Veh, 10+LY: 0.93 ± 0.14, *P < 0.05 vs* 10+Veh: 0.71 ± 0.07; Fig. 1F, cell permeability, x-fold compared with 5+Veh, 10+LY: 1.11 ± 0.56, *P < 0.05 vs* 10+Veh: 3.09 ± 1.32). Immunofluorescence staining shows that 10 % stretch significantly disrupted cell-cell junctions formed by VE-cadherin, which was prevented by LY-333531 (Fig. 1G). These data are in line with our previous findings that EMPA prevents the cell permeability disruption induced by 10 % stretch [9]. The endothelial protective effect of LY-333531 was not further amplified when LY-333531 was combined with EMPA, suggestive of similar mechanisms of protection by these two compounds (Figs. 1E and 10+EMPA + LY: 0.93 ± 0.18. *P > 0.05 vs* 10+LY; Figs. 1F and 10+EMPA + LY: 1.09 ± 0.27, *P > 0.05 vs* 10+LY). Taken together, EMPA reduces increased ROS production and cell permeability in 10%-stretched endothelial cells by inhibiting PKC activity.

Furthermore, 10 nM PKC activator PMA stimulated ROS production in ECs undergoing 5 % stretch, suggesting PKC as one of the pivotal mediators for oxidative stress in HCAECs (Fig. S1). Application of GKT136901 completely abrogated the increase in NOX activity measured from cell lysates, proving NOX1/4 as the dominant isoform that contribute to NOX activation in stretched ECs (Fig. S2). The sensitivity of applied NOX activity was validated by a linear regression analysis between the crude protein amount and measured NADH consumption rate (Fig. S2). Application of EMPA and LY-333531 alone did not modulate ROS production in HCAECs exposed to 5 % stretch (Fig. S3, *P* both *>0.05*).

### 3.2 PKC knockdown prevents the increase in NOX activity and ROS generation in HCAECs exposed to Ca^2+^ ionophore ionomycin

**Fig. 2:**
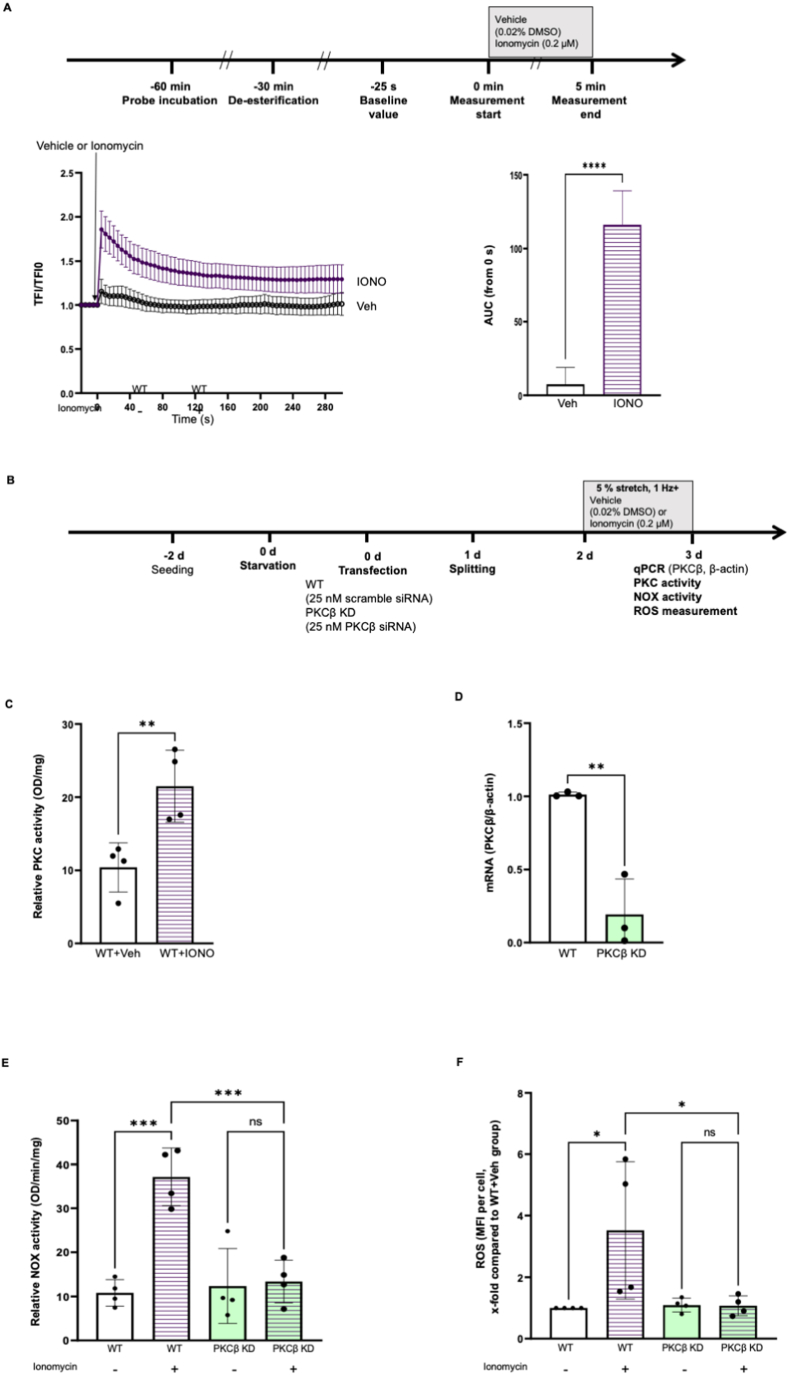
**PKC knockdown prevents the increase in NOX activity and ROS generation in HCAECs exposed to Ca**^**2+**^**ionophore ionomycin.** Cells were incubated with Fluo4-AM for 30 min, followed by 30 min de-esterification, Ca^2+^ was measured using live cell imaging. Briefly, cells were imaged every 5 s for five times to obtain a baseline value of total fluorescence intensity (TFI0). Afterwards, 0.2 μM ionomycin or 0.02 % DMSO (Veh) was loaded on the cells and the changes of fluorescence intensities of cells were recorded every 5 s for 5 min. The increase in intracellular Ca^2+^ was quantified using the area under curve (AUC) (A, n = 5). Representative images are shown in Figs. S16–A. To explore the role of PKC-β in Ca^2+^ related oxidative stress, PKC-β knock down was performed using siRNA. Wilde-type HCAECs or PKC-β knockdown cells were subjected to 5 % stretch for 24 h with or without 0.2 μM ionomycin, the study flowchart is shown in Fig. 3B. PKC activity was measured from wild-type cells receiving vehicle or ionomycin (D, n = 4). The efficiency of PKC-β knockdown was validated with qPCR (D, n = 3). NOX activity was measured from cell lysates (E, n = 4) and ROS were quantified using mean fluorescence intensity (MFI) (F, n = 4). Data are presented as mean ± SD. *P < 0.05*, referred as *; ** for *P < 0.01*, *** for *P < 0.001* and **** for *P < 0.0001*, were considered statistically significant. ROS data were normalized to 5+Veh group and non-normalized ROS values were presented in Fig. S10 and Table S2.

Calcium overload directly activates PKC-β by recruiting its C2 domain to the cellular membrane, therefore promoting the development of pro-arrhythmogenic cardiac alternants in rabbit hearts [23,24]. To explore the functional relationship between cytosolic Ca^2+^ and oxidative stress in HCAECs, 0.2 μM ionomycin (IONO) was applied to enhance intracellular Ca^2+^ (Fig. 2A, AUC, Veh: 7.59 ± 5.09 vs IONO: 116.10 ± 10.28, *P < 0.05*). Additionally, ionomycin potently activated PKC in endothelial cells (Fig. 2C). Transfection with specific siRNA reduced PKC-β expression by 80 % (Fig. 2D, PKC-β/β-actin, WT: 1.01 ± 0.02 vs PKC-β KD: 0.19 ± 0.24, *P < 0.05*), which potently abrogated the increase in NOX activity and ROS production in HCAECs cells receiving ionomycin (Fig. 2E, NOX activity, OD/mg/min, WT + Veh: 10.83 ± 3.02, *P < 0.05* vs WT + IONO: 37.13 ± 6.55; PKC-β KD + Veh: 12.37 ± 8.47, *P > 0.05* vs PKC-β KD + IONO: 13.39 ± 4.85; Fig. 2F, ROS, x-fold compared with PKC-β KD + IONO: 1.07 ± 0.32, *P < 0.05* vs WT + IONO: 3.52 ± 2.23; PKC-β KD + Veh: 1.09 ± 0.23, *P > 0.05* vs PKC-β KD + IONO). Furthermore, PKC-β knockdown blocked the NOX activation by 10 % stretch (Fig. S4), indicating PKC-β as a mediator for stretch related oxidative stress.

### 3.3 EMPA might reduce intracellular Ca^2+^ through NCX, leading to the inhibition of PKC activation and oxidative stress in 10%-stretched HCAECs

**Fig. 3:**
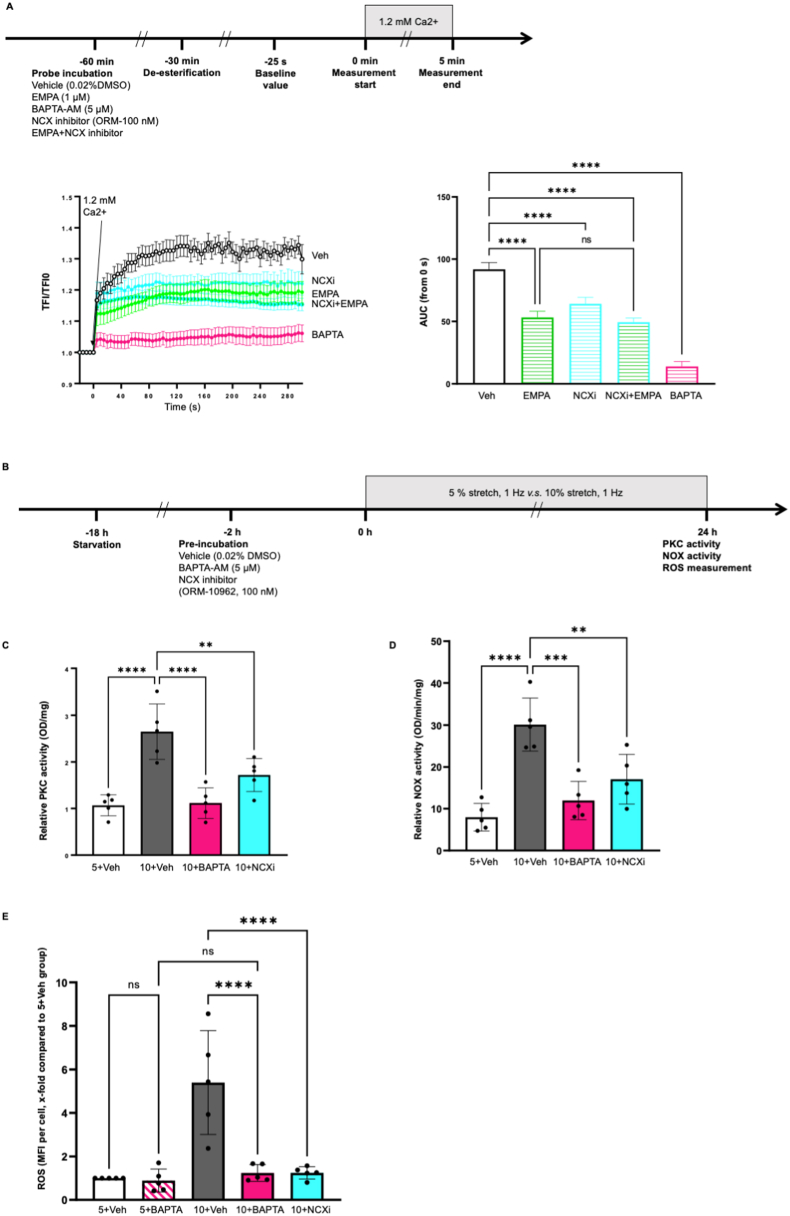
**EMPA reduces intracellular Ca**^**2+**^**probably through NCX, leading to the inhibition of PKC activation and oxidative stress in 10%-stretched HCAECs.** Cells were incubated with Fluo4-AM for 30 min in the presence of either vehicle, 1 μM EMPA, 100 nM ORM-10962, EMPA plus ORM-10962, or 5 μM BAPTA-AM, followed by 30 min de-esterification with respective treatment, and Ca^2+^ was measured using live cell imaging. Briefly, cells were imaged every 5 s for five times to get a baseline value of total fluorescence intensity (TFI0). Afterwards, 1.2 mM Ca^2+^ was loaded on the cells and the changes of fluorescence intensities of cells were recorded every 5 s for 5 min. The increase in intracellular Ca^2+^ was quantified using the area under curve (AUC) (Figure A, n = 5). Representative images are shown in Figs. S16–B. To investigate the casual link between NCX/Ca^2+^ and stretch related endothelial dysfunction, cells were pre-incubated for 2 h with vehicle, 100 nM ORM-10962, or 5 μM BAPTA-AM, before exposure to 5 % stretch or 10 % stretch. After 24 h, PKC activity, NOX activity and ROS were measured from cell lysate (C, n = 5; D, n = 5), the flow chart is shown in Fig. 3B. ROS were quantified using mean fluorescence intensity (MFI) (E, n = 5). Data are presented as mean ± SD. *P < 0.05*, referred as *; ** for *P < 0.01*, *** for *P < 0.001* and **** for *P < 0.0001*, were considered statistically significant. ROS data were normalized to the 5+Veh group and non-normalized ROS values are presented in Fig. S11 and Table S2.

**Fig. 4:**
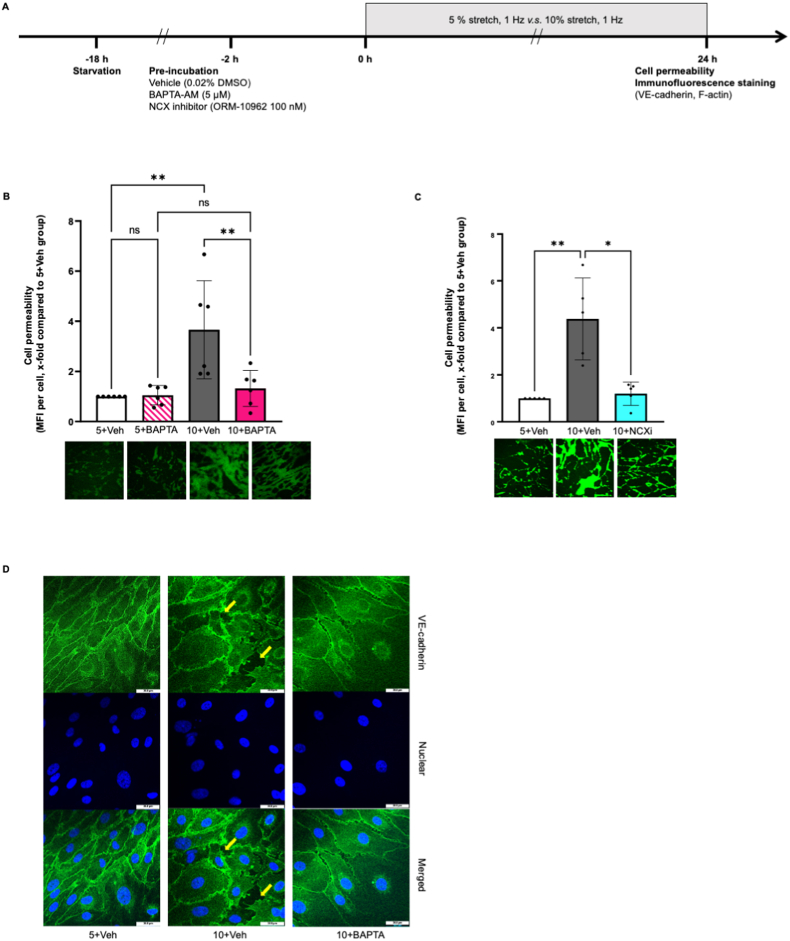
**BAPTA-AM and ORM-10962 prevent the enhanced cell permeability in HCAECs undergoing 10 % stretch.** Cells were pre-incubated for 2 h with vehicle, 100 nM ORM-10962, or 5 μM BAPTA-AM, before exposure to 5 % stretch or 10 % stretch. After 24 h, cell permeability assay and VE-cadherin staining were performed and the experiment flow chart is shown in Fig. 4A. Cell permeability was quantified using mean fluorescence intensity (MFI) and all the data were normalized to 5+Veh group (B, n = 6; C, n = 5). Representative images for VE-cadherin staining are shown in Fig. 4D. Data are presented as mean ± SD. *P < 0.05*, referred as *; ** for *P < 0.01*, *** for *P < 0.001* and **** for *P < 0.0001*, were considered statistically significant.

In static HCAECs, EMPA, NCX inhibitor ORM-10962 and calcium chelator BAPTA-AM lowered intracellular Ca^2+^ in HCAECs (Fig. 3A, AUC, EMPA: 53.23 ± 4.94, NCXi: 64.17 ± 5.11, BAPTA: 13.90 ± 3.94, *P* all *<0.05 vs* Veh: 91.86 ± 5.41). Combination of EMPA and NCX inhibitor ORM-10962 did not cause further reduction in Ca^2+^ when compared to EMPA alone (Fig. 3A, NCXi + EMPA: 49.40 ± 3.37, *P > 0.05 vs* EMPA), suggesting that EMPA might lower Ca^2+^ in HCAECs via inhibition of NCX. In cells exposed to 10 % stretch, BAPTA-AM and ORM-10962 both prevented the enhanced PKC activity and oxidative stress caused by 10 % stretch (Fig. 3C, OD/mg, 10+BAPTA: 1.11 ± 0.33, 10+NCXi: 1.72 ± 0.35, *P* both*<0.05 vs* 10+Veh: 2.65 ± 0.59; Fig. 3D, NOX activity, OD/mg/min, 5+Veh: 7.96 ± 3.29, 10+BAPTA: 11.96 ± 4.58, 10+NCXi: 17.06 ± 5.95, *P* all *<0.05 vs* 10+Veh: 30.13 ± 6.34; Fig. 3E, ROS, x-fold compared with 5+Veh, 10+BAPTA: 1.24 ± 0.39, 10+NCXi: 1.25 ± 0.28, *P* both*<0.05 vs* 10+Veh: 5.05 ± 2.30). Additionally, BAPTA-AM and ORM-10962 both restored the increased cell permeability of cells undergoing 10 % stretch BAPTA-AM and ORM-10962 both restored the increased cell permeability of cells undergoing 10 % stretch (Fig. 4B, cell permeability, x-fold compared with 5+Veh, 10+BAPTA: 1.32 ± 0.72 *vs* 10+Veh: 3.66 ± 1.96; Fig. 4C, cell permeability, x-fold compared with 5+Veh, 10+NCXi: 1.20 ± 0.50 *vs* 10+Veh: 4.38 ± 1.74, *P* both*<0.05*). BAPTA-AM prevented the VE-cadherin disruption in 10%-stretched HCAECs (Fig. 4D), suggesting that intracellular Ca^2+^ is one intermediator for stretch related endothelial barrier dysfunction [25,26].

To further validate the role of NCX1 as an intermediator for stretch related PKC activation, activity of PKC was measured from NCX1 siRNA knockdown HCAECs undergoing 24 h stretch. Data from three individual experiments suggested that knockdown of NCX1 prevented the increase in PKC activity caused by 10 % stretch (Fig. S5).

### 3.4 EMPA inhibits stretch induced PKC activity and NOX activation via inhibition of the NHE/Na^+^ pathway

**Fig. 5:**
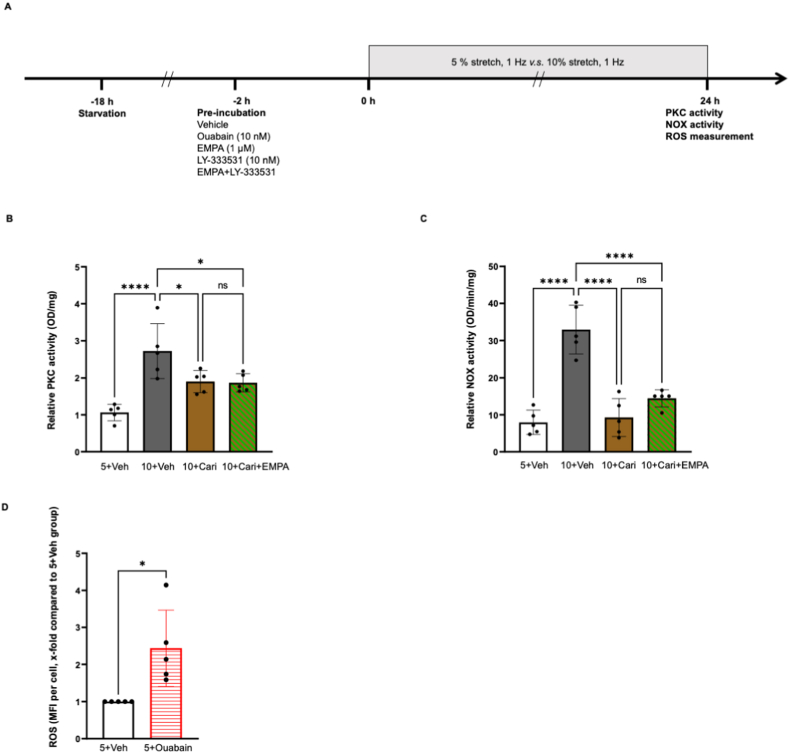
**EMPA inhibits stretch increased PKC activity and NOX activation via the NHE/Na** + **pathway.** Cells were pre-incubated with either vehicle, 10 μM cariporide, or cariporide plus 1 μM EMPA before exposure to 5 % or 10 % stretch, 10 nM ouabain was applied on cells undergoing 5 % stretch to investigate the functional link between increased Na^+^ and oxidative stress. The flow chart is shown in Fig. 5A. PKC activity and NOX activity were detected at 24 h from cells treated with cariporide or combined treatment (B, n = 5; C, n = 5). Influence of ouabain on ROS production was analysed from cells exposure to physiological stretch (D, n = 5). Data are presented as mean ± SD. *P < 0.05*, referred as *; ** for *P < 0.01*, *** for *P < 0.001* and **** for *P < 0.0001*, were considered statistically significant. ROS data were normalized to 5+Veh group and non-normalized ROS values are presented in Fig. S12 and Table S2.

The NHE inhibitor cariporide prevented the increase in PKC activity induced by 10 % stretch (Fig. 5B, OD/mg, 5+Veh: 1.07 ± 0.23, 10+Cari: 1.90 ± 0.30, *P* both *<0.05 vs* 10+Veh: 2.72 ± 0.74), and the PKC inhibitory effect of cariporide was not augmented when combined with EMPA (Figs. 5B and 10+Cari + EMPA: 1.87 ± 0.25, *P > 0.05 vs* 10+Cari). Combination of cariporide and EMPA exerted similar inhibitory capacity on stretch enhanced NOX activity as cariporide alone (Fig. 5C, relative NOX activity, OD/min/mg, 10+Cari: 9.26 ± 5.15 vs 10+Veh: 32.95 ± 6.57, *P < 0.05*; 10+Cari + EMPA: 14.47 ± 2.32, *P > 0.05 vs* 10+Cari). Additional experiments revealed that ouabain potently upregulated ROS generation in HCAECs exposed to 5 % stretch (Fig. 5D, x-fold compared with 5+Veh, 5+ouabain: 2.44 ± 1.03, *P < 0.05* vs 5+Veh).

### 3.5 EMPA reduces intracellular Ca^2+^ via Na^+^/NCX pathway, which is partially mediated by the inhibition of NHE

**Fig. 6:**
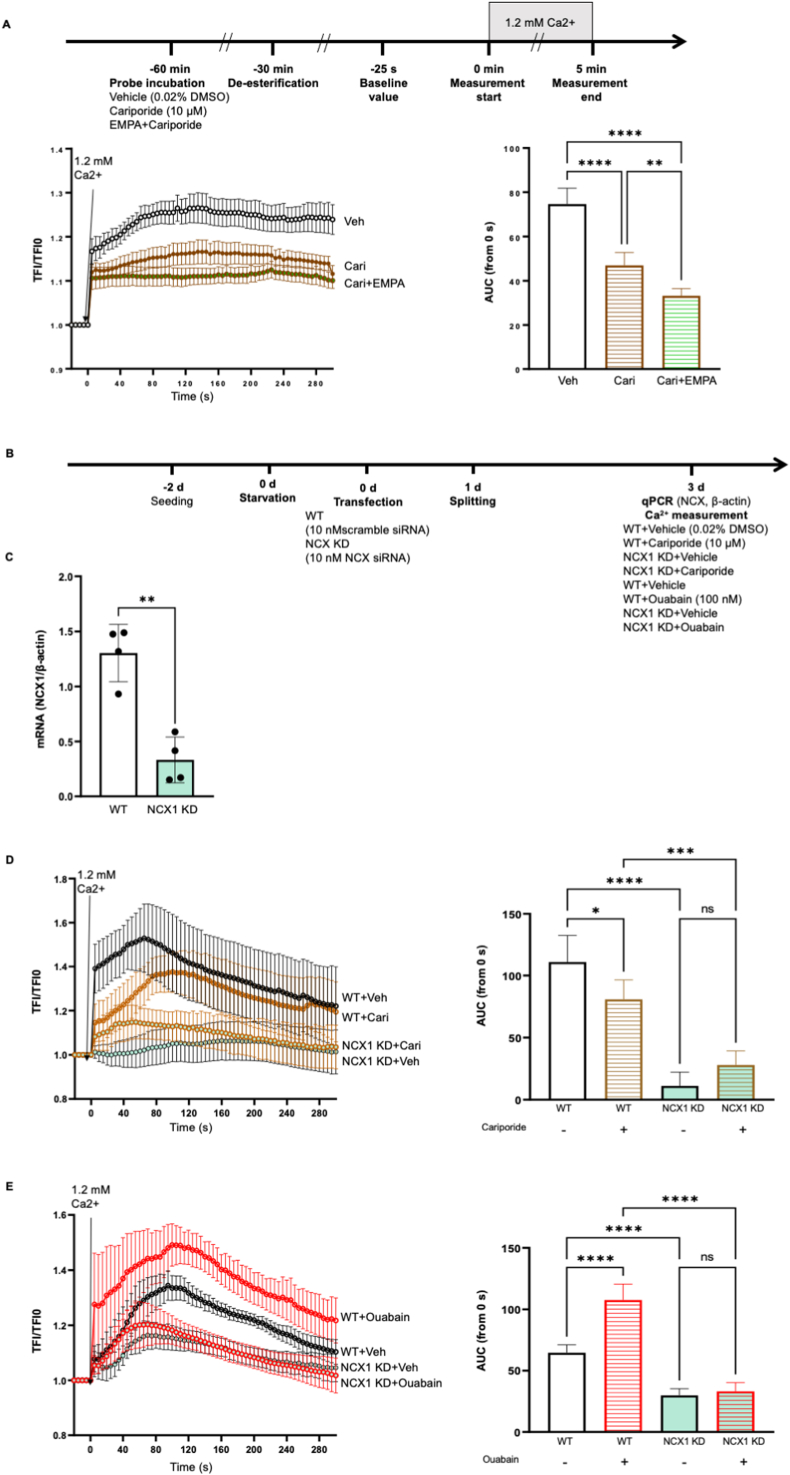
**EMPA reduces intracellular Ca**^**2+**^**via the Na**^**+**^**/NCX pathway, which is partially mediated by the inhibition of NHE.** Cells were incubated with Fluo4-AM for 30 min in the presence of either vehicle, 10 μM cariporide, or cariporide plus 1 μM EMPA, followed by 30 min de-esterification, Ca^2+^ was measured using live cell imaging. The increase in intracellular Ca^2+^ was quantified using the area under curve (AUC) (A, n = 5). Representative images are shown in Figs. S16–C. To show the interaction between NHE1/Na^+^ and NCX1 in the Ca^2+^ mobilization of HCAECs, Ca^2+^ measurements were performed in cells transfected with NCX1 siRNA, in the presence of cariporide or ouabain (C, n = 5; D, n = 5). The flow chart is presented in Fig. 6B and representative images are shown in Figure S16-D and S16-E, respectively. NCX1 knockdown was validated using qPCR (C, n = 4). Data are presented as mean ± SD. *P < 0.05*, referred as *; ** for *P < 0.01*, *** for *P < 0.001* and **** for *P < 0.0001*, were considered statistically significant.

In the current study, we investigated if EMPA reduces intracellular Ca^2+^ through inhibiting NHE activity and lowering cytosolic Na^+^ in HCAECs. The NHE inhibitor cariporide reduced intracellular Ca^2+^ in resting ECs (Fig. 6A, AUC, Cari: 46.95 ± 5.84, *P < 0.05 vs* Veh: 74.64 ± 7.15), revealing a causal link between lowered NHE activity and decreased intracellular Ca^2+^ in human ECs. Surprisingly, the combination of cariporide with EMPA exerted an even more potent Ca^2+^ inhibition than cariporide alone (Cari + EMPA: 33.26 ± 3.24, *P < 0.05 vs* Cari).

**Fig. 7:**
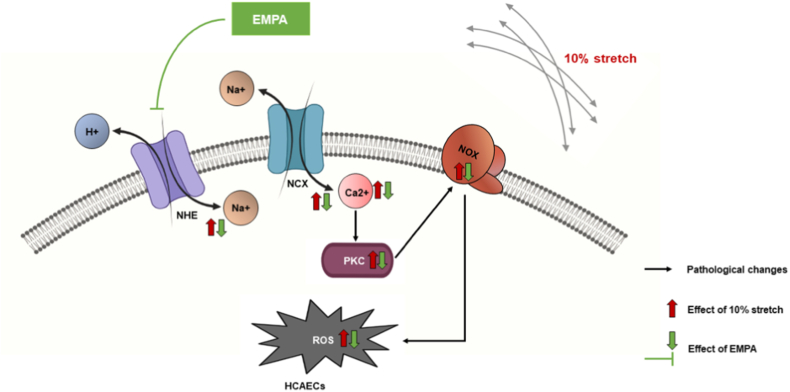
Synopsis of the main outcomes of the study. EMPA lowers intracellular Ca^2+^ via inhibiting the NHE/Na^+^/NCX axis, in turn preventing PKC activity and NOX activation induced by 10 % stretch, as well as suppressing ROS generation.

In order to explore the interaction of NHE1, cytosolic Na^+^ and NCX1 in the Ca^2+^ mobilization of HCAECs, Ca^2+^ measurements were performed in NCX1 knockdown cells in the presence of cariporide or ouabain. NCX knockdown led to further Ca^2+^ decrease in HCAECs receiving cariporide (Fig. 6D, AUC, WT + Cari: 87.77 ± 15.71, *P < 0.05 vs* NCX1 KD + Cari: 28.0 ± 11.22), while the NHE inhibitor did not reduce the intracellular Ca^2+^ in NCX inhibited cells (NCX1 KD + Veh:11.10 ± 11.16, *P > 0.05 vs* NCX1 KD + Cari). Additionally, NCX knockdown completely abrogated the enhancement in intracellular Ca^2+^ caused by the sodium pump inhibitor ouabain (Fig. 6E, AUC, WT + Veh: 64.77 ± 6.40, *P < 0.05 vs* WT + Ouabain: 107.60 ± 12.97; NCX1 KD + Veh: 29.75 ± 5.583, *P > 0.05 vs* WT + Ouabain: 33.19 ± 7.115), showing that accumulated cytosolic Na ^+^ can trigger the increase in intracellular Ca^2+^ in HCAECs through NCX. Taken together, these data suggest that EMPA might lower cytosolic Ca^2+^ through inhibition of the Na^+^/NCX axis, and this effect is partially mediated by NHE. The proposed mechanism behind the anti-oxidative effect of EMPA in cells exposed to 10 % stretch is summarized in Fig. 7.

Noticeably, the current manuscript showed no significant effect of 10 % stretch or EMPA on the mRNA and protein levels of PKC-β, NOX4, NCX1 and NHE1, suggesting that the endothelial protective effect of EMPA is not mediated by changes in the expression of these genes (Figs. S6–S8).

## 4 Discussion

The major findings of the present study are: (1) EMPA prevents NOX activation and ROS production by inhibiting PKC activity in HCAECs exposed to 10 % stretch; and (2) the PKC suppression by EMPA is mediated through inhibition of the NHE/Na^+^/NCX/Ca^2+^ axis.

### 4.1 EMPA ameliorates stretch related endothelial dysfunction by inhibition of PKC

In stretched human urothelial cells, PKC α, β and ζ are translocated from cytosol (quiescent location) to nuclei and cellular membranes (active location), suggesting a potential activation of these isoforms by stretch [27]. Correspondingly, we detected an induction of PKC activity in HCAECs exposed to 10 % stretch. The increase in PKC activity was significantly reverted by EMPA, revealing a novel PKC inhibitory effect of this SGLT2i in stretched HCAECs. EMPA and the PKC inhibitor LY-333531 both prevented stretch-induced NOX activation and ROS production, showing an essential involvement of PKC in the anti-oxidative effect of EMPA.

Hyperglycaemia might enhance total PKC activity, and consequently compromises barrier integrity of human brain microvascular endothelial cells [13]. More recently, O'Neil et al. demonstrated that the PKC activator PMA stimulates NOX4 expression and ROS generation in cord blood-derived endothelial colony-forming cells, therefore promoting their *in vitro* migration and tubulogenesis capacity [28]. Inhibition of NOX4 by VAS2870 abolished the PMA-related tube formation, highlighting the interaction between PKC and NOX in the maintenance of cardiovascular haemostasis [28].

However, the involvement of individual PKC isoforms in the development of cardiovascular dysfunction is still a matter of debate. According to our current study, specific blockage of PKC-β by 10 nM LY-333531 reverted the oxidative stress and the barrier dysfunction of pathologically stretched HCAECs, and these effects were not further amplified when combined with EMPA. Moreover, PKC-β knockdown completely abrogated the NOX activation in HCAECs undergoing 10 % stretch. These data suggest that PKC-β is the major mediator of the endothelial protection by EMPA under stretch conditions. Correspondingly, a previous study showed that 400 nM LY-333531 (resembling an all-PKC isoforms inhibiting concentration) does not further prevent hyperglycaemia-induced monocyte attachment to human ECs when compared with 10 nM LY-33351 (a PKC-β specific inhibiting concentration) [29], further showing the dominant role of PKC-β activation in endothelial dysfunction. However, inhibition of PKC-α and -β isoforms were both shown to counteract the deterioration of human cerebral barrier by hyperglycaemia [13].

### 4.2 Inhibition of stretch induced PKC activity by EMPA is mediated via NHE/Na^+^/NCX/Ca^2+^ axis

EMPA lowered intracellular Ca^2+^ concentration in isolated ventricular myocytes from rats and rabbits [14]. More recently, Mustroph et al. showed that 24 h exposure to 1 μM EMPA strongly reduces sarcoplasmic reticulum Ca^2+^ leakage in failing cardiomyocytes of human and mice [30], suggesting that Ca^2+^ inhibition is a piece of the puzzle underlying cardiovascular protection by EMPA. Increased intracellular Ca^2+^ is an indispensable regulator of PKC-β activity, one of the classical PKC isoforms that contain a calcium-binding C2 domain. Upon exposure to increased Ca^2+^, PKC-β is activated following the binding of Ca^2+^ to its C2 domain, the translocation to the cellular membrane, and the interaction between C1a domain and its ligand diacyglycerol [31]. In the current study, a knockdown of PKC-β by 70 % blocks the increased NOX activity and ROS production induced by ionomycin, suggesting a causal relation between Ca^2+^-dependent PKC activation and oxidative stress in HCAECs. Furthermore, we explored the effect of EMPA on cytosolic Ca^2+^ in HCAECs. In line with previous findings in cardiomyocytes, we found that 1 μM EMPA, as well as BAPTA-AM, lowers cytosolic Ca^2+^ in HCAECs. Additionally, BAPTA-AM prevents the induction of PKC activation, oxidative stress and cell permeability by increased stretch, indicating that Ca^2+^ is involved in the protective effect of EMPA against stretch-related endothelial dysfunction.

Lowering intracellular Ca^2+^ by EMPA in cardiomyocytes occurs secondary to the reduction in intracellular Na^+^, which might be mediated by the direct NHE inhibitory capacity of EMPA [14]. In the present study we focused on the detailed molecular mechanisms underlying the Ca^2+^ lowering effect of EMPA. Inhibition of NCX by ORM-10962 and NCX1 knockdown both reduced intracellular Ca^2+^ concentration in HCAECs, and the combination of EMPA and an NCX inhibitor does not lead to further reduction compared to ORM-10962 alone. These data suggest NCX1 as a potential mediator for the Ca^2+^ reducing effect of EMPA. Furthermore, we observed increased cytosolic Ca^2+^ levels in HCAECs following exposure to the sodium pump inhibitor ouabain, as well as a decrease in intracellular Ca^2+^ levels in cells treated with the NHE inhibitor cariporide. Moreover, NCX inhibition/knockdown and cariporide attenuated the increased PKC activity in HCAECs exposed to 10 % stretch, while ouabain stimulated oxidative stress in cells undergoing 5 % stretch. These data indicate the involvement of NHE, Na^+^ and NCX in the stretch related PKC activation and oxidative stress of HCAECs. To further explore the interaction between NCX1 and NHE/Na^+^ in the Ca^2+^ mobilization in endothelial cells, Ca^2+^ measurements were performed in cells transfected with NCX1 siRNA. NCX1 knockdown abolishes the Ca^2+^ enhancing effect of ouabain, showing that NCX1 is a crucial mediator for the increase in cytosolic Ca^2+^ secondary to Na ^+^ accumulation.

Noticeably, combination of EMPA with cariporide demonstrated an additional inhibition on Ca^2+^ enhancement compared to cariporide alone. The NHE inhibitory capacity of EMPA is still a matter of debate. In contrast to our findings, Juni et al. reported a more effective ROS reduction by 1 μM EMPA than by 10 μM cariporide (63 % vs. 38 %) in microvascular ECs stimulated by uremic serum from patients with chronic kidney disease [32]. This study indicated that at least part of the endothelial protection via EMPA does not involve NHE. Moreover, NCX1 knockdown further lowers intracellular Ca^2+^ in HCAECs receiving cariporide, while cariporide does not reduce the cytosolic Ca^2+^ in NCX1 knockdown cells. These data indicate that the Ca^2+^ reducing effect of NHE inhibitor cariporide is mediated by NCX1, but only part of the Ca^2+^ mobilization through NCX1 happens secondarily to NHE inhibition. More recent studies suggest that EMPA also lowers intracellular Na^+^ in cardiomyocytes through direct or indirect inhibition of the late sodium channel current (e.g., through Ca^2+^/Calmodulin-dependent kinase II) [33,34]. Yet, it is still unclear if late sodium channel is expressed in human ECs. These novel findings concerning the inhibitory effects of EMPA on ion channels other than NHE might also help to interpret the present data. Furthermore, another structure-based computation study has been performed to compare the ability of EMPA and cariporide to bind to NHE [35]. EMPA provides an even more stable interaction with NHE than cariporide, which might explain the stronger NHE-mediated Ca^2+^ reduction by EMPA compared to cariporide [35].

### 4.3 Limitations

The current project is an *in vitro* study performed with HCAECs isolated from healthy donors. Whether the findings reported here can be translated to patients with cardiovascular disease requires further investigation. Cyclic stretch was induced using the FX-6000T^TM^ Tension System. It is not feasible to conduct real-time live cell imaging on this device using inverted Microscopy, because a non-transparent baseplate beneath the cell culture wells is needed during stretch. Thus, all calcium measurements in our study were performed using static cells. Nevertheless, considering that EMPA strongly reduces intracellular Ca^2+^ concentration, and that the calcium chelator BAPTA-AM significantly attenuate PKC activation and oxidative stress in ECs subjected to 10 % stretch, we have confirmed our hypothesis that the protective effect of EMPA on pathologically stretched ECs is mediated by Ca^2+^ related pathways. Furthermore, there is still an ongoing debate about the involvement of SGLT2 in the endothelial effects of EMPA. However, our recent study showed that EMPA demonstrates comparable protective effect against ischemia/reperfusion induced heart infarction in wild-type mice and global SGLT2-knockout mice, providing for the first time a strong evidence for the SGLT2-independent cardiovascular benefit of EMPA [36]. We did not measure the direct effect of EMPA on NHE activity and intracellular Na^+^ as we have previously shown that EMPA potently lowers NHE activity and cytosolic Na^+^ in HCAECs [10]. We have demonstrated in numerous publications that EMPA potently inhibited NHE activity in various cell types such as human endothelial cells, rabbit cardiomyocytes, mouse cardiomyocytes, and demonstrated by in silico NHE-docking studies the binding of SGLT2i’s to NHE1 [12,37]. NHE1 inhibition by SGLT2i’s has also been demonstrated for human atrial cardiomyocyte cells [38]. Three different laboratories around the world have demonstrated, using various techniques, that EMPA directly binds to the NHE [35,39,40]. These findings have provided robust evidence that EMPA inhibits and binds to the NHE in various cell types. Yet, it is true that the precise molecular binding partners (amino acids) of SGLT2i’s to NHE1 are still unclear and should be resolved, but we consider this as outside the scope of the current work. Using the well-reported intracellular Na^+^ enhancer ouabain and NHE inhibitor cariporide [10,26], this study has given a detailed insight into the NHE/Na^+^/NCX1 interaction in the endothelial protective effects of EMPA. Additionally, the current manuscript suggested that stretch or EMPA did not influence the mRNA levels of target genes (e.g., PKCβ, NHE1). Despite unchanged RNA quantities, post-transcriptional and post-translational mechanisms could also lead to variations in protein levels. Further studies are required to explore whether changes in protein activities in HCAECs exposed to 10 % stretch or EMPA are based on alterations in mRNA quantity, protein quantity, or interactions of the enzyme with activators or inhibitors.

## 5 Conclusion

We here demonstrated a novel PKC inhibitory effect of EMPA in HCAECs under mechanical forces, which may also be a major pathway to explain the observed anti-oxidative effects of SGLT2i’s on cells exposed to different pathological stimuli (e.g. hyperglycaemia, inflammatory cytokines, cyclic stretch). Using mechanically activated HCAECs, we further showed that modulation of intracellular ion homeostasis (e.g. Na^+^ and Ca^2+^) by EMPA through ion channels like NHE and NCX contributes to the amelioration of endothelial dysfunction. These findings improve the understanding of the cardiovascular benefits of SGLT2i’s.

## Funding

X.L. and M.W. are supported by Chinese Scholarship Council (CSC) fellowship program (2019xxxx0054 and 2022xxxx0005). The research received no specific grant from any funding agency in the public, commercial or non-for-profit sectors.

## Author contribution

**Xiaoling Li**: Data acquisition and analysis, Data interpretation, Manuscript preparation and editing; **Mengnan Wang**: Data acquisition and analysis, Data interpretation; **Jan-Ole Kalina**: Data acquisition and analysis, Data interpretation; **Benedikt Preckel**: Conceptualization, Data interpretation, Manuscript preparation and editing; **Markus W. Hollmann**: Conceptualization, Data interpretation, Manuscript editing; **Martin Albrecht**: Conceptualization, Data interpretation, Manuscript editing; **Coert J. Zuurbier**: Conceptualization, Data interpretation, Manuscript editing; **Nina C. Weber**: Conceptualization, Data interpretation, Manuscript preparation and editing, Supervision.

## Declaration of competing interest

Authors declare that there are no conflicts of interest.
