# Supplementary material for: Empagliflozin prevents oxidative stress in human coronary artery endothelial cells via the NHE/PKC/NOX axis
Source: Redox Biol. 2023 Dec 2;69:102979. doi: 10.1016/j.redox.2023.102979 (PMC10749278; doi:10.1016/j.redox.2023.102979)
Supplement: Multimedia component 1 [file mmc1.pptx]

## Slide 1
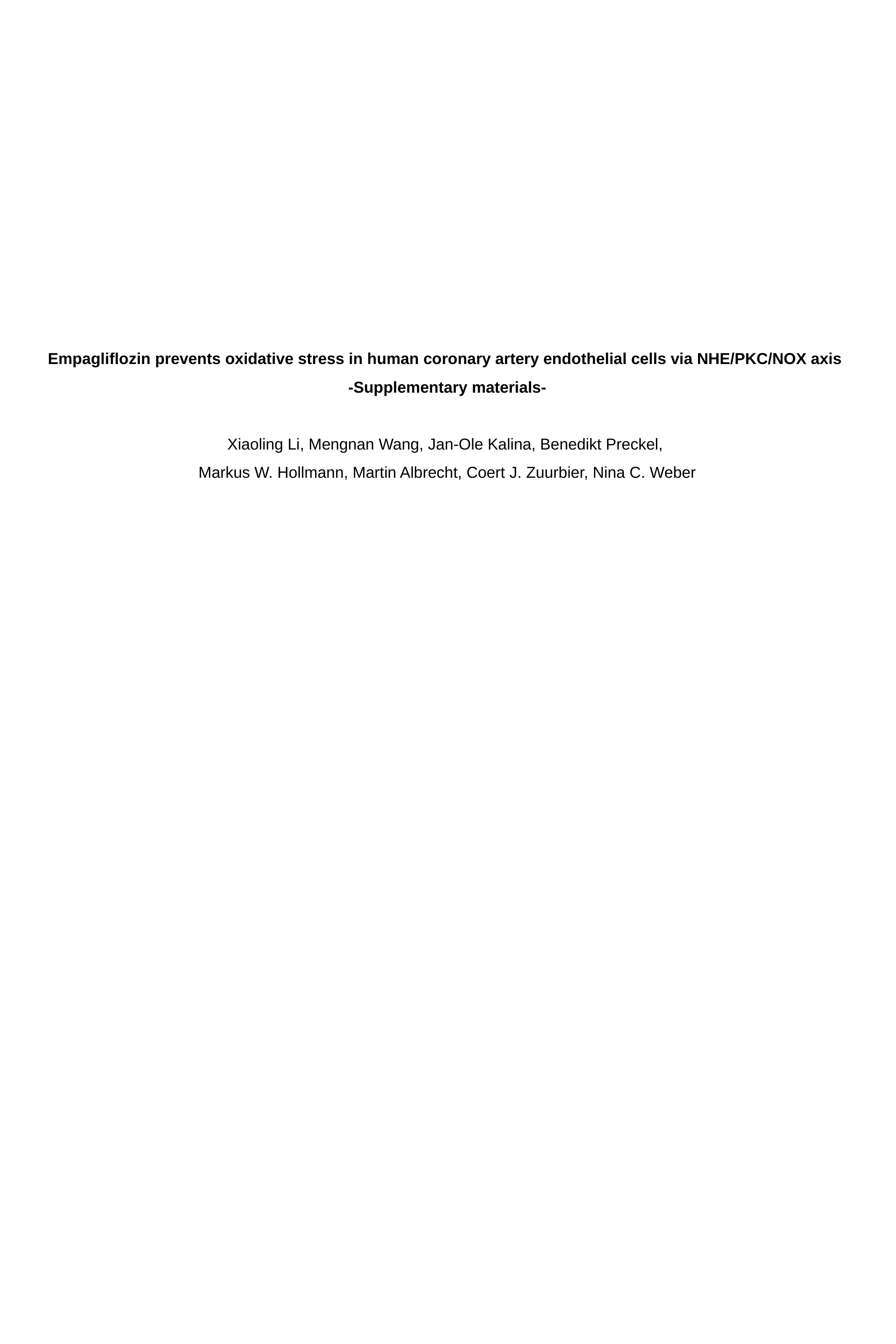

Empagliflozin prevents oxidative stress in human coronary artery endothelial cells via NHE/PKC/NOX axis
-Supplementary materials-
Xiaoling Li, Mengnan Wang, Jan-Ole Kalina, Benedikt Preckel,
Markus W. Hollmann, Martin Albrecht, Coert J. Zuurbier, Nina C. Weber

## Slide 2
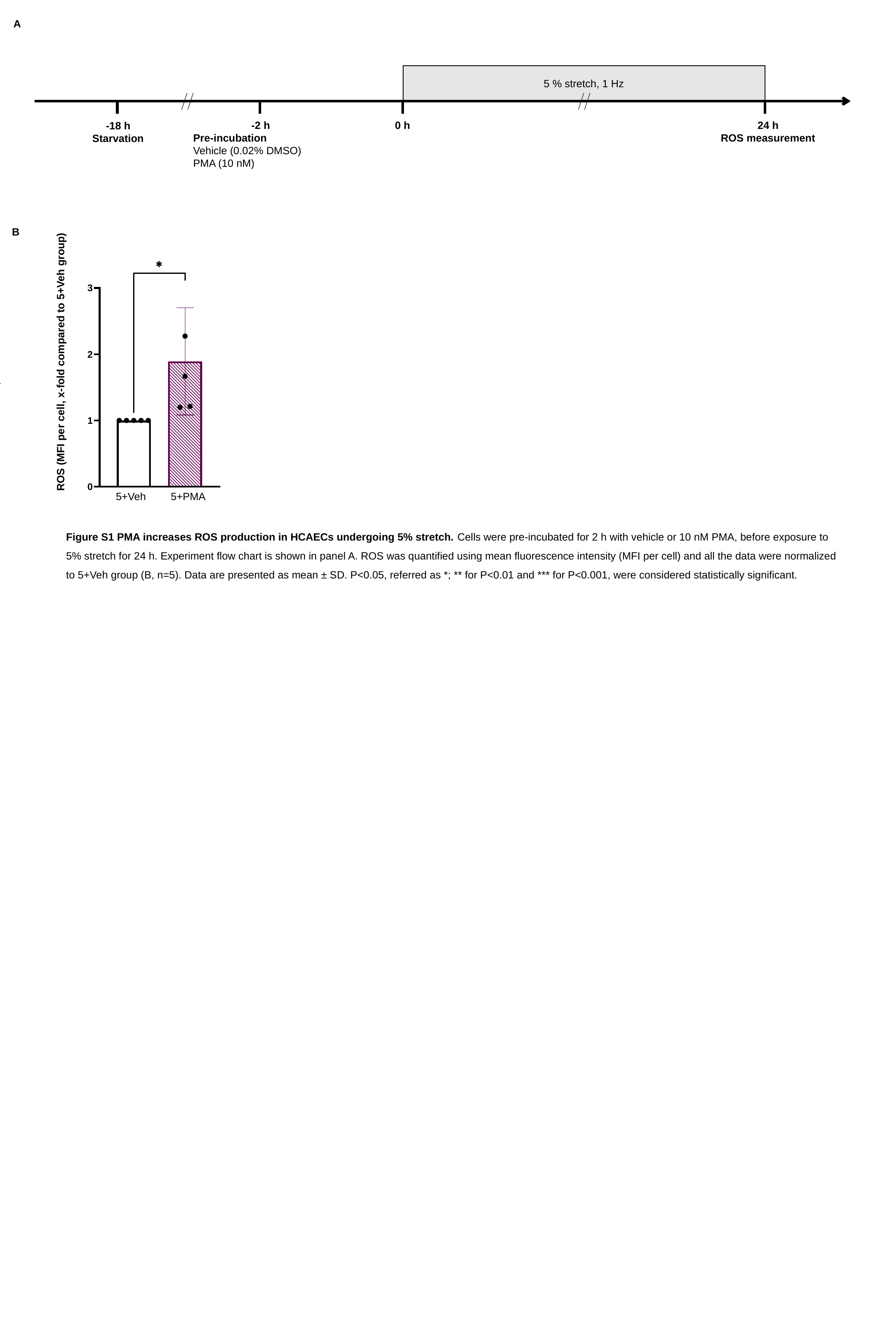

A
5 % stretch, 1 Hz
-2 h
Pre-incubation
Vehicle (0.02% DMSO)
PMA (10 nM)
0 h
24 h
ROS measurement
-18 h
Starvation
B
ROS (MFI per cell, x-fold compared to 5+Veh group)
5+Veh
5+PMA
Figure S1 PMA increases ROS production in HCAECs undergoing 5% stretch. Cells were pre-incubated for 2 h with vehicle or 10 nM PMA, before exposure to 5% stretch for 24 h. Experiment flow chart is shown in panel A. ROS was quantified using mean fluorescence intensity (MFI per cell) and all the data were normalized to 5+Veh group (B, n=5). Data are presented as mean ± SD. P<0.05, referred as *; ** for P<0.01 and *** for P<0.001, were considered statistically significant.

## Slide 3
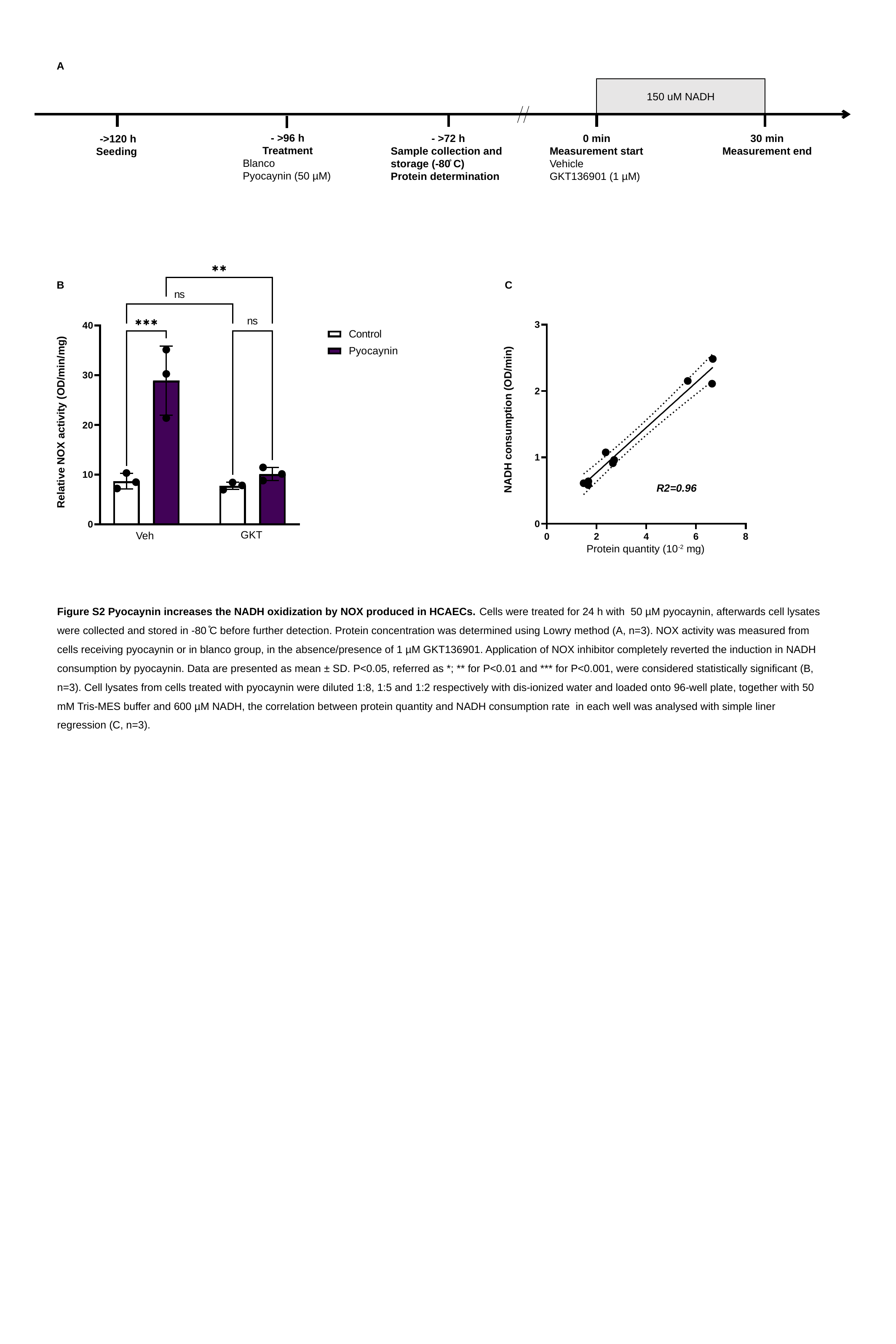

A
150 uM NADH
- >72 h
Sample collection and storage (-80̊ C)
Protein determination
0 min
Measurement start
Vehicle
GKT136901 (1 µM)
30 min
Measurement end
->120 h
Seeding
- >96 h
Treatment
Blanco
Pyocaynin (50 µM)
Relative NOX activity (OD/min/mg)
GKT
Veh
B
C
NADH consumption (OD/min)
Protein quantity (10-2 mg)
R2=0.96
Figure S2 Pyocaynin increases the NADH oxidization by NOX produced in HCAECs. Cells were treated for 24 h with 50 µM pyocaynin, afterwards cell lysates were collected and stored in -80 ̊C before further detection. Protein concentration was determined using Lowry method (A, n=3). NOX activity was measured from cells receiving pyocaynin or in blanco group, in the absence/presence of 1 µM GKT136901. Application of NOX inhibitor completely reverted the induction in NADH consumption by pyocaynin. Data are presented as mean ± SD. P<0.05, referred as *; ** for P<0.01 and *** for P<0.001, were considered statistically significant (B, n=3). Cell lysates from cells treated with pyocaynin were diluted 1:8, 1:5 and 1:2 respectively with dis-ionized water and loaded onto 96-well plate, together with 50 mM Tris-MES buffer and 600 µM NADH, the correlation between protein quantity and NADH consumption rate in each well was analysed with simple liner regression (C, n=3).

## Slide 4
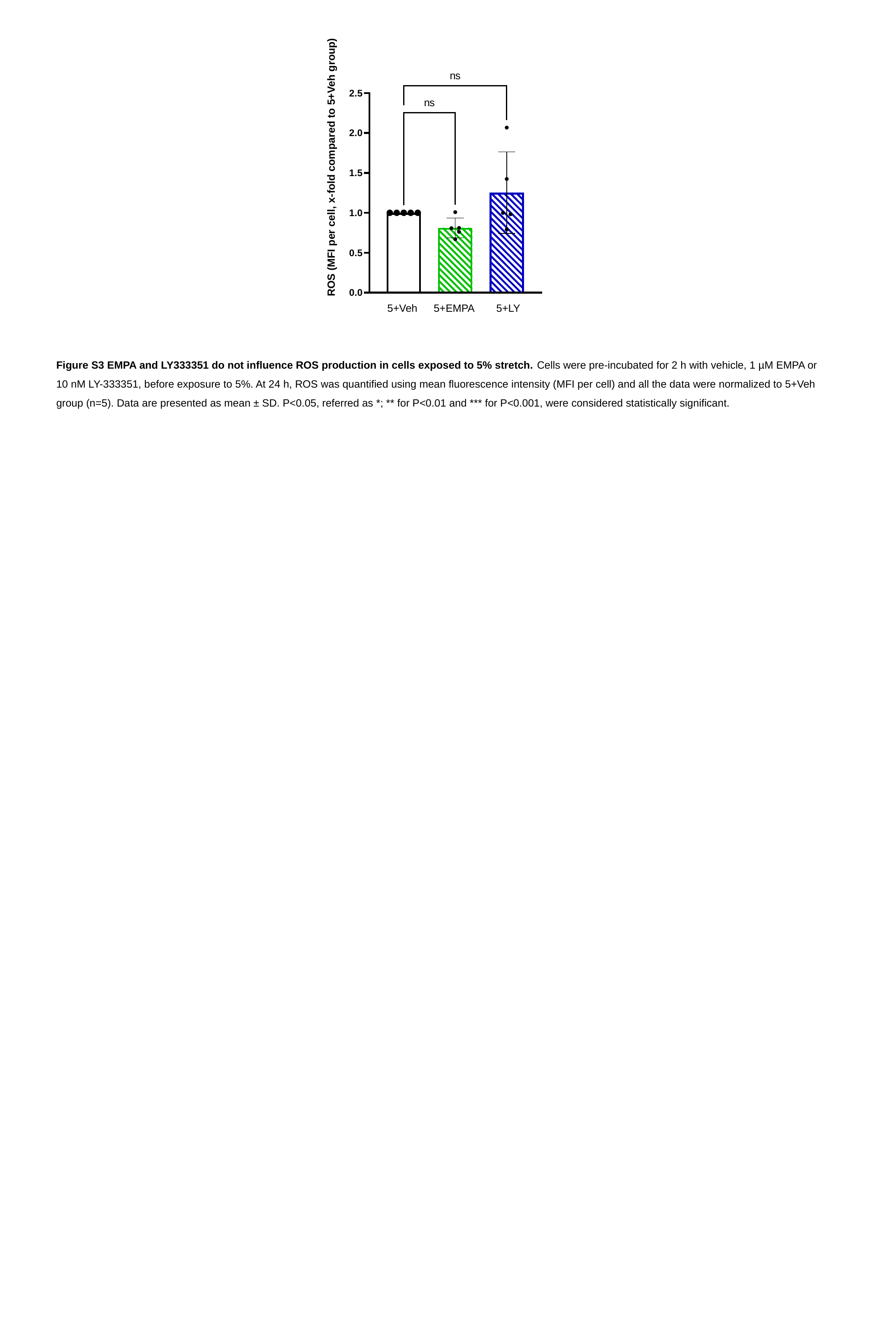

ROS (MFI per cell, x-fold compared to 5+Veh group)
5+Veh
5+EMPA
5+LY
Figure S3 EMPA and LY333351 do not influence ROS production in cells exposed to 5% stretch. Cells were pre-incubated for 2 h with vehicle, 1 µM EMPA or 10 nM LY-333351, before exposure to 5%. At 24 h, ROS was quantified using mean fluorescence intensity (MFI per cell) and all the data were normalized to 5+Veh group (n=5). Data are presented as mean ± SD. P<0.05, referred as *; ** for P<0.01 and *** for P<0.001, were considered statistically significant.

## Slide 5
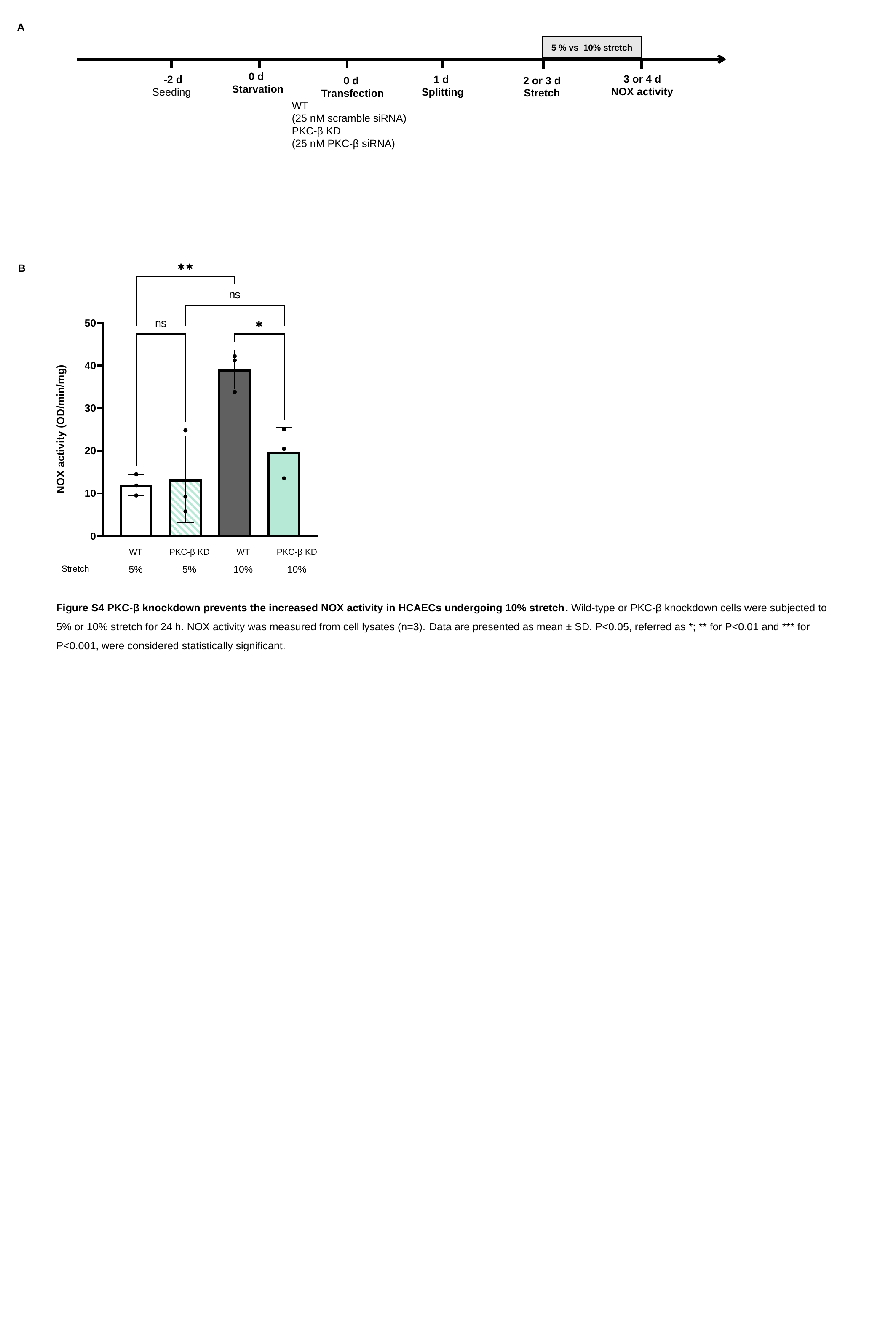

A
5 % vs 10% stretch
0 d
Starvation
3 or 4 d
NOX activity
1 d
Splitting
 -2 d
Seeding
0 d
Transfection
WT
(25 nM scramble siRNA)
PKC-β KD
(25 nM PKC-β siRNA)
2 or 3 d
Stretch
B
NOX activity (OD/min/mg)
| | WT | PKC-β KD | WT | PKC-β KD |
| --- | --- | --- | --- | --- |
| Stretch | 5% | 5% | 10% | 10% |
Figure S4 PKC-β knockdown prevents the increased NOX activity in HCAECs undergoing 10% stretch. Wild-type or PKC-β knockdown cells were subjected to 5% or 10% stretch for 24 h. NOX activity was measured from cell lysates (n=3). Data are presented as mean ± SD. P<0.05, referred as *; ** for P<0.01 and *** for P<0.001, were considered statistically significant.

## Slide 6
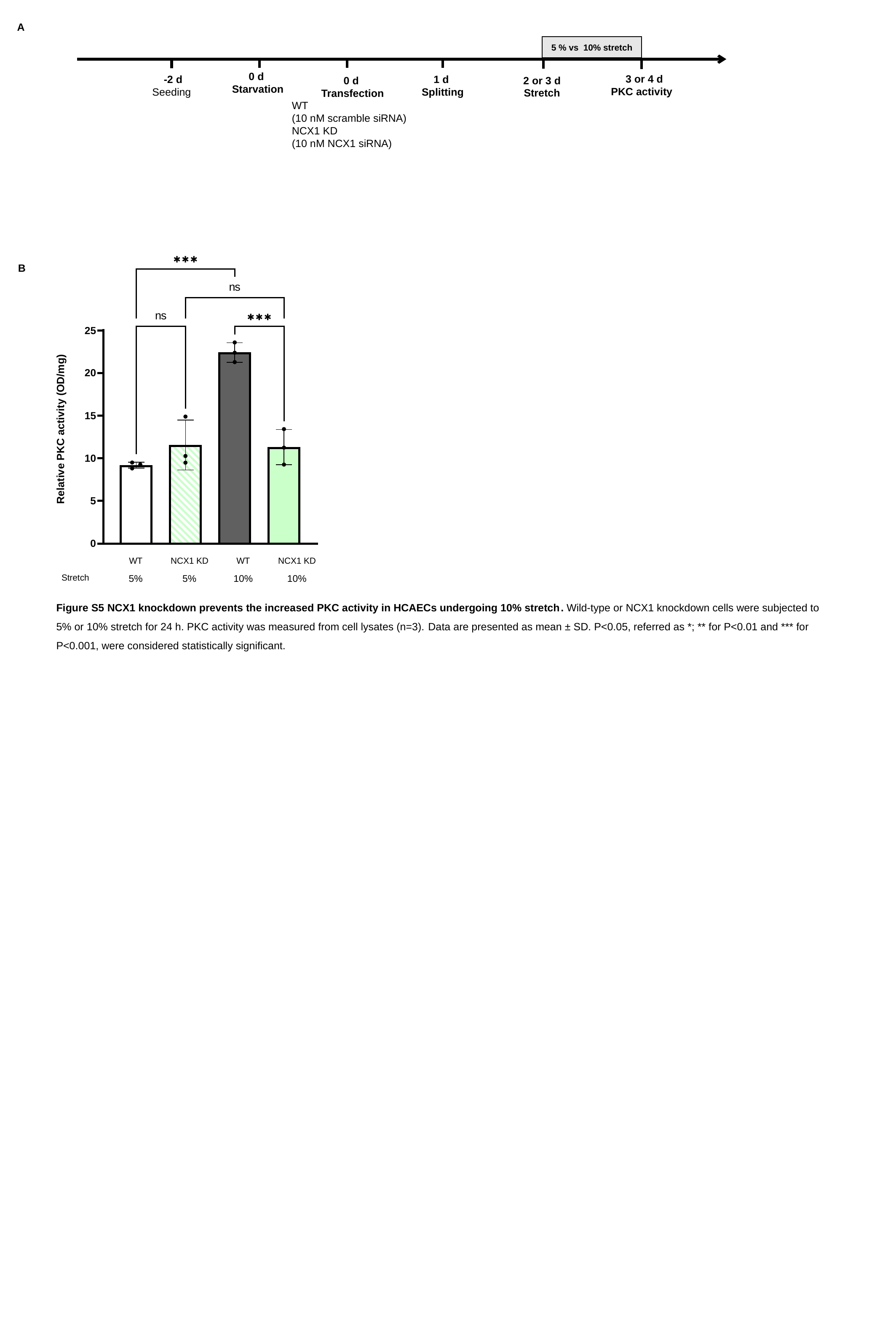

A
5 % vs 10% stretch
0 d
Starvation
3 or 4 d
PKC activity
1 d
Splitting
 -2 d
Seeding
0 d
Transfection
WT
(10 nM scramble siRNA)
NCX1 KD
(10 nM NCX1 siRNA)
2 or 3 d
Stretch
B
Relative PKC activity (OD/mg)
B
| | WT | NCX1 KD | WT | NCX1 KD |
| --- | --- | --- | --- | --- |
| Stretch | 5% | 5% | 10% | 10% |
Figure S5 NCX1 knockdown prevents the increased PKC activity in HCAECs undergoing 10% stretch. Wild-type or NCX1 knockdown cells were subjected to 5% or 10% stretch for 24 h. PKC activity was measured from cell lysates (n=3). Data are presented as mean ± SD. P<0.05, referred as *; ** for P<0.01 and *** for P<0.001, were considered statistically significant.

## Slide 7
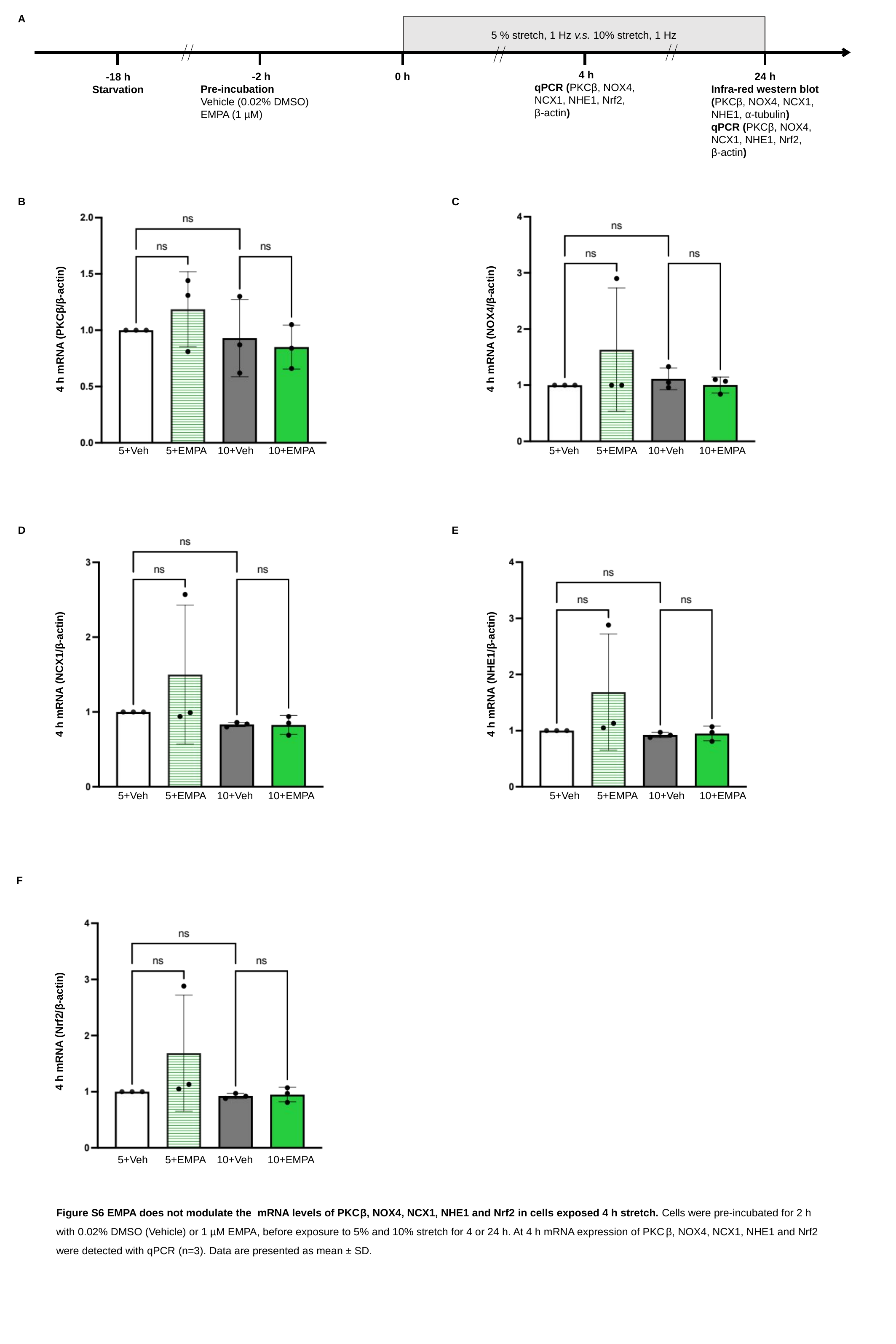

A
5 % stretch, 1 Hz v.s. 10% stretch, 1 Hz
-2 h
Pre-incubation
Vehicle (0.02% DMSO)
EMPA (1 µM)
0 h
24 h
Infra-red western blot
(PKCβ, NOX4, NCX1,
NHE1, α-tubulin)
qPCR (PKCβ, NOX4,
NCX1, NHE1, Nrf2,
β-actin)
-18 h
Starvation
4 h
qPCR (PKCβ, NOX4,
NCX1, NHE1, Nrf2,
β-actin)
B
C
4 h mRNA (PKCβ/β-actin)
5+Veh
5+EMPA
10+Veh
10+EMPA
4 h mRNA (NOX4/β-actin)
5+Veh
5+EMPA
10+Veh
10+EMPA
D
E
4 h mRNA (NCX1/β-actin)
5+Veh
5+EMPA
10+Veh
10+EMPA
4 h mRNA (NHE1/β-actin)
5+Veh
5+EMPA
10+Veh
10+EMPA
F
4 h mRNA (Nrf2/β-actin)
5+Veh
5+EMPA
10+Veh
10+EMPA
Figure S6 EMPA does not modulate the mRNA levels of PKCβ, NOX4, NCX1, NHE1 and Nrf2 in cells exposed 4 h stretch. Cells were pre-incubated for 2 h with 0.02% DMSO (Vehicle) or 1 µM EMPA, before exposure to 5% and 10% stretch for 4 or 24 h. At 4 h mRNA expression of PKCβ, NOX4, NCX1, NHE1 and Nrf2 were detected with qPCR (n=3). Data are presented as mean ± SD.

## Slide 8
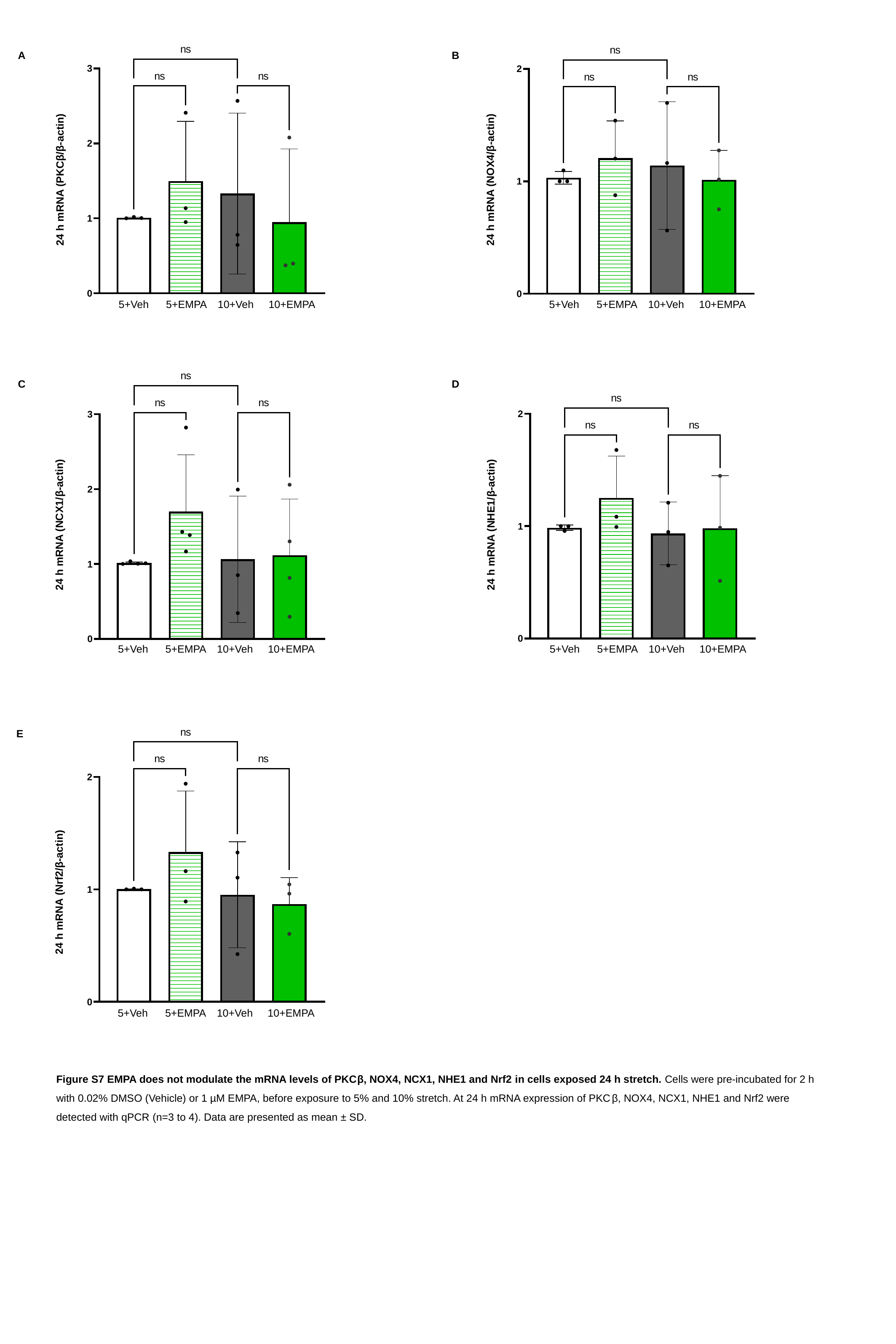

A
B
24 h mRNA (PKCβ/β-actin)
5+Veh
5+EMPA
10+Veh
10+EMPA
24 h mRNA (NOX4/β-actin)
5+Veh
5+EMPA
10+Veh
10+EMPA
C
D
24 h mRNA (NCX1/β-actin)
5+Veh
5+EMPA
10+Veh
10+EMPA
24 h mRNA (NHE1/β-actin)
5+Veh
5+EMPA
10+Veh
10+EMPA
E
24 h mRNA (Nrf2/β-actin)
5+Veh
5+EMPA
10+Veh
10+EMPA
Figure S7 EMPA does not modulate the mRNA levels of PKCβ, NOX4, NCX1, NHE1 and Nrf2 in cells exposed 24 h stretch. Cells were pre-incubated for 2 h with 0.02% DMSO (Vehicle) or 1 µM EMPA, before exposure to 5% and 10% stretch. At 24 h mRNA expression of PKCβ, NOX4, NCX1, NHE1 and Nrf2 were detected with qPCR (n=3 to 4). Data are presented as mean ± SD.

## Slide 9
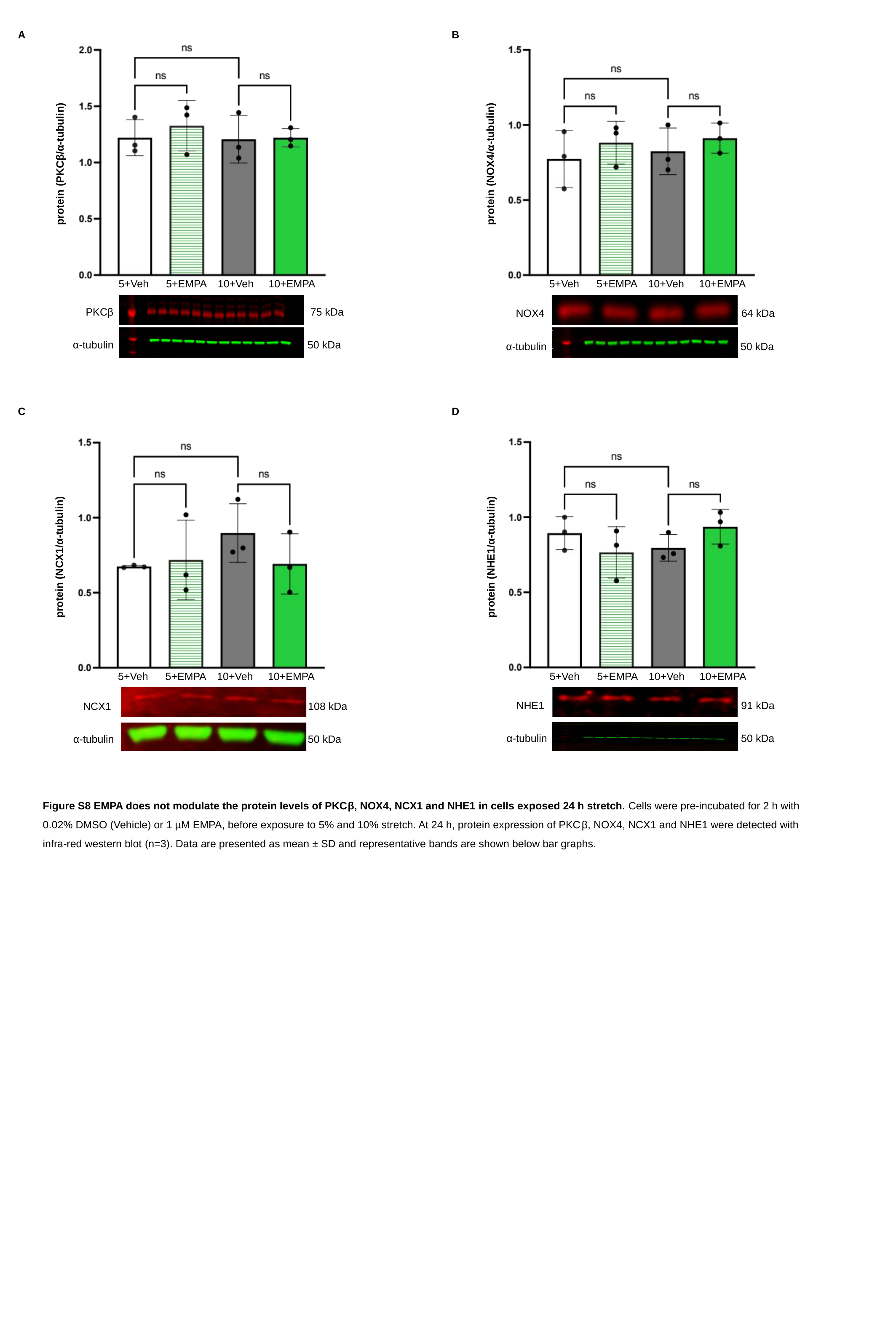

A
B
protein (PKCβ/α-tubulin)
5+Veh
5+EMPA
10+Veh
10+EMPA
protein (NOX4/α-tubulin)
5+Veh
5+EMPA
10+Veh
10+EMPA
PKCβ 75 kDa
NOX4 64 kDa
α-tubulin 50 kDa
α-tubulin 50 kDa
C
D
protein (NCX1/α-tubulin)
5+Veh
5+EMPA
10+Veh
10+EMPA
protein (NHE1/α-tubulin)
5+Veh
5+EMPA
10+Veh
10+EMPA
NHE1 91 kDa
NCX1 108 kDa
α-tubulin 50 kDa
α-tubulin 50 kDa
Figure S8 EMPA does not modulate the protein levels of PKCβ, NOX4, NCX1 and NHE1 in cells exposed 24 h stretch. Cells were pre-incubated for 2 h with 0.02% DMSO (Vehicle) or 1 µM EMPA, before exposure to 5% and 10% stretch. At 24 h, protein expression of PKCβ, NOX4, NCX1 and NHE1 were detected with infra-red western blot (n=3). Data are presented as mean ± SD and representative bands are shown below bar graphs.

## Slide 10
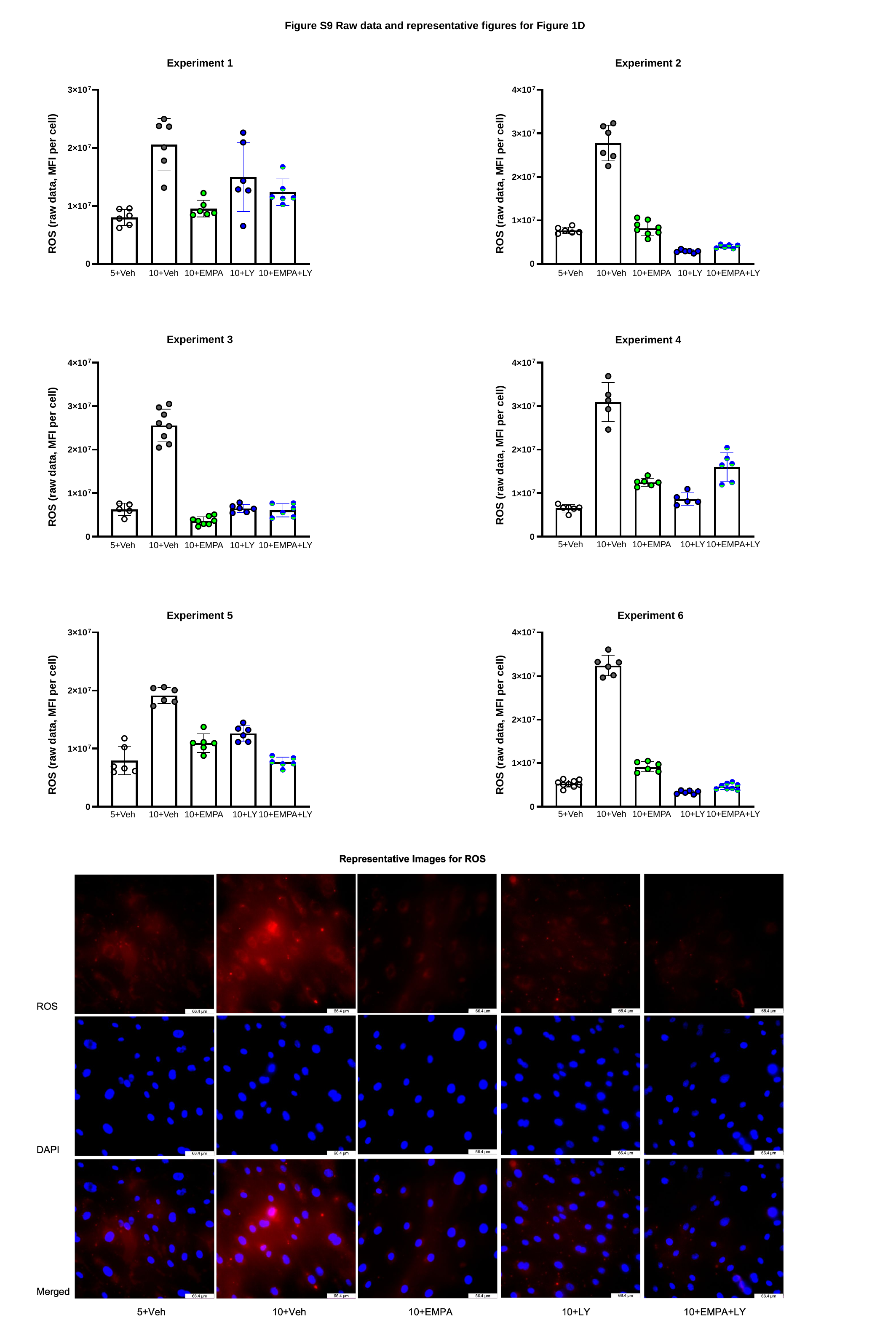

Figure S9 Raw data and representative figures for Figure 1D
Experiment 1
Experiment 2
ROS (raw data, MFI per cell)
5+Veh
10+Veh
10+EMPA
10+LY
10+EMPA+LY
ROS (raw data, MFI per cell)
5+Veh
10+Veh
10+EMPA
10+LY
10+EMPA+LY
Experiment 3
Experiment 4
ROS (raw data, MFI per cell)
5+Veh
10+Veh
10+EMPA
10+LY
10+EMPA+LY
ROS (raw data, MFI per cell)
5+Veh
10+Veh
10+EMPA
10+LY
10+EMPA+LY
Experiment 6
Experiment 5
ROS (raw data, MFI per cell)
5+Veh
10+Veh
10+EMPA
10+LY
10+EMPA+LY
ROS (raw data, MFI per cell)
5+Veh
10+Veh
10+EMPA
10+LY
10+EMPA+LY

## Slide 11
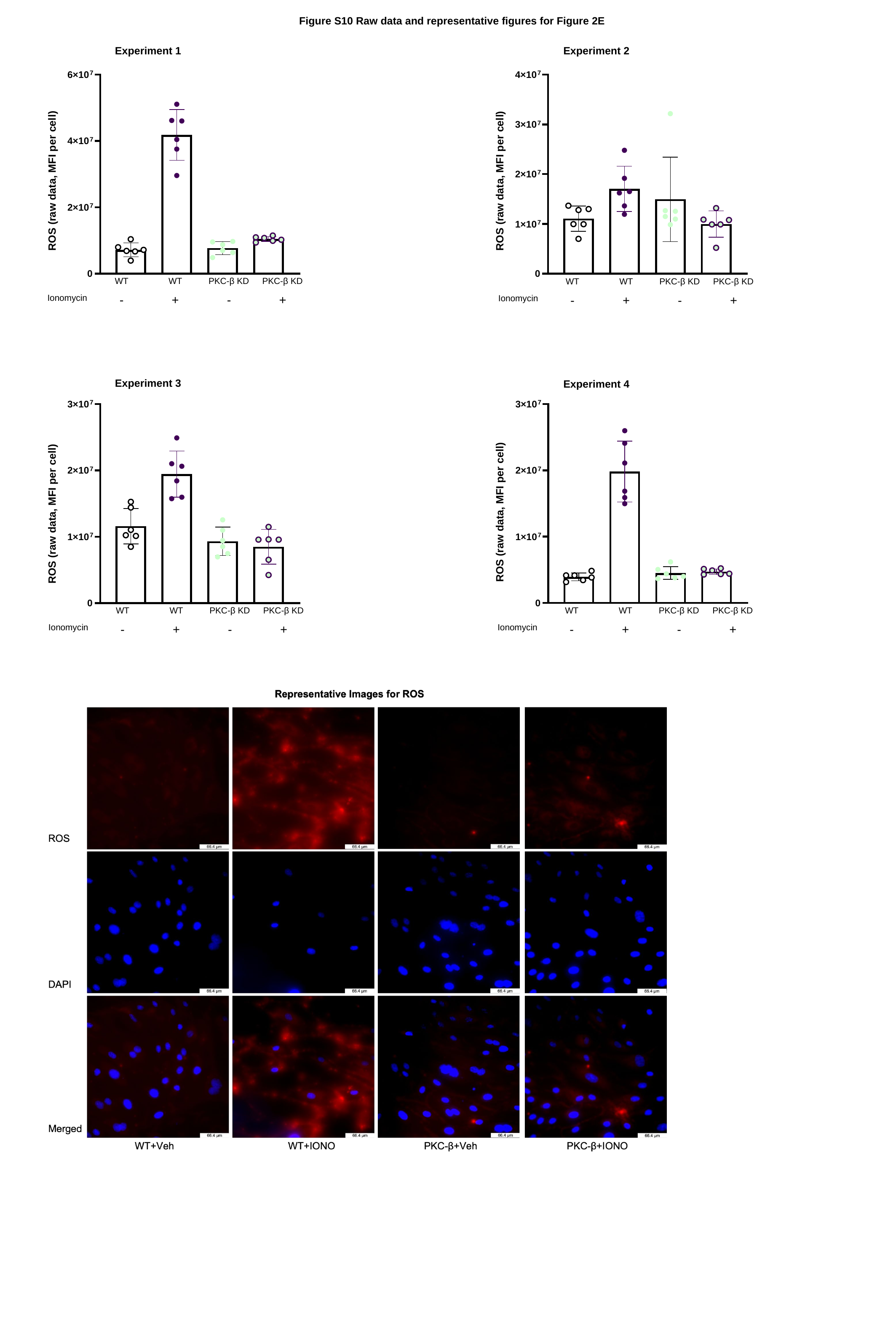

Figure S10 Raw data and representative figures for Figure 2E
Experiment 1
Experiment 2
ROS (raw data, MFI per cell)
ROS (raw data, MFI per cell)
| | WT | WT | PKC-β KD | PKC-β KD |
| --- | --- | --- | --- | --- |
| Ionomycin | - | + | - | + |
| | WT | WT | PKC-β KD | PKC-β KD |
| --- | --- | --- | --- | --- |
| Ionomycin | - | + | - | + |
Experiment 3
Experiment 4
ROS (raw data, MFI per cell)
ROS (raw data, MFI per cell)
| | WT | WT | PKC-β KD | PKC-β KD |
| --- | --- | --- | --- | --- |
| Ionomycin | - | + | - | + |
| | WT | WT | PKC-β KD | PKC-β KD |
| --- | --- | --- | --- | --- |
| Ionomycin | - | + | - | + |

## Slide 12
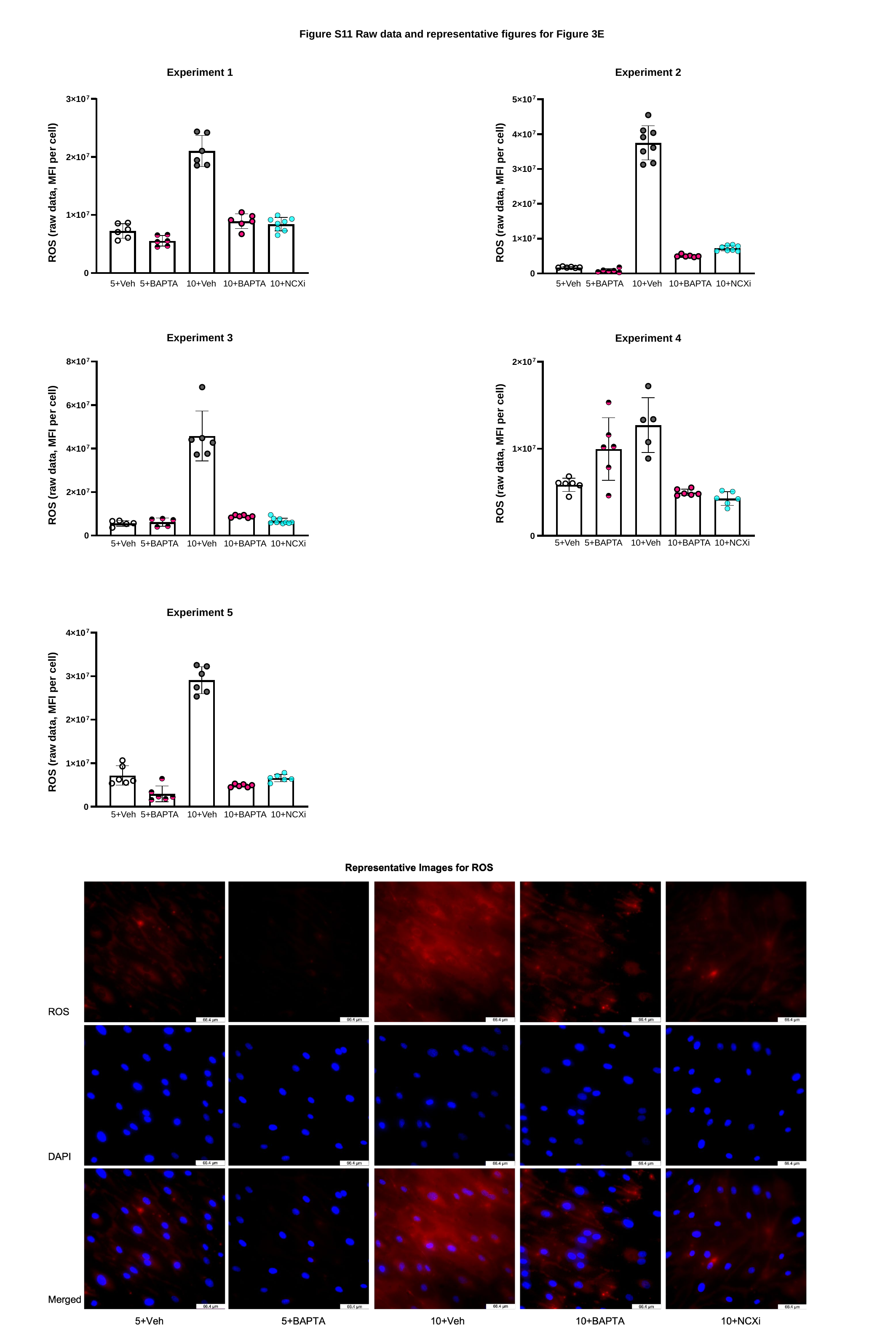

Figure S11 Raw data and representative figures for Figure 3E
Experiment 1
Experiment 2
ROS (raw data, MFI per cell)
ROS (raw data, MFI per cell)
5+Veh
5+BAPTA
10+Veh
10+BAPTA
10+NCXi
5+Veh
5+BAPTA
10+Veh
10+BAPTA
10+NCXi
Experiment 3
Experiment 4
ROS (raw data, MFI per cell)
ROS (raw data, MFI per cell)
5+Veh
5+BAPTA
10+Veh
10+BAPTA
10+NCXi
5+Veh
5+BAPTA
10+Veh
10+BAPTA
10+NCXi
Experiment 5
ROS (raw data, MFI per cell)
5+Veh
5+BAPTA
10+Veh
10+BAPTA
10+NCXi

## Slide 13
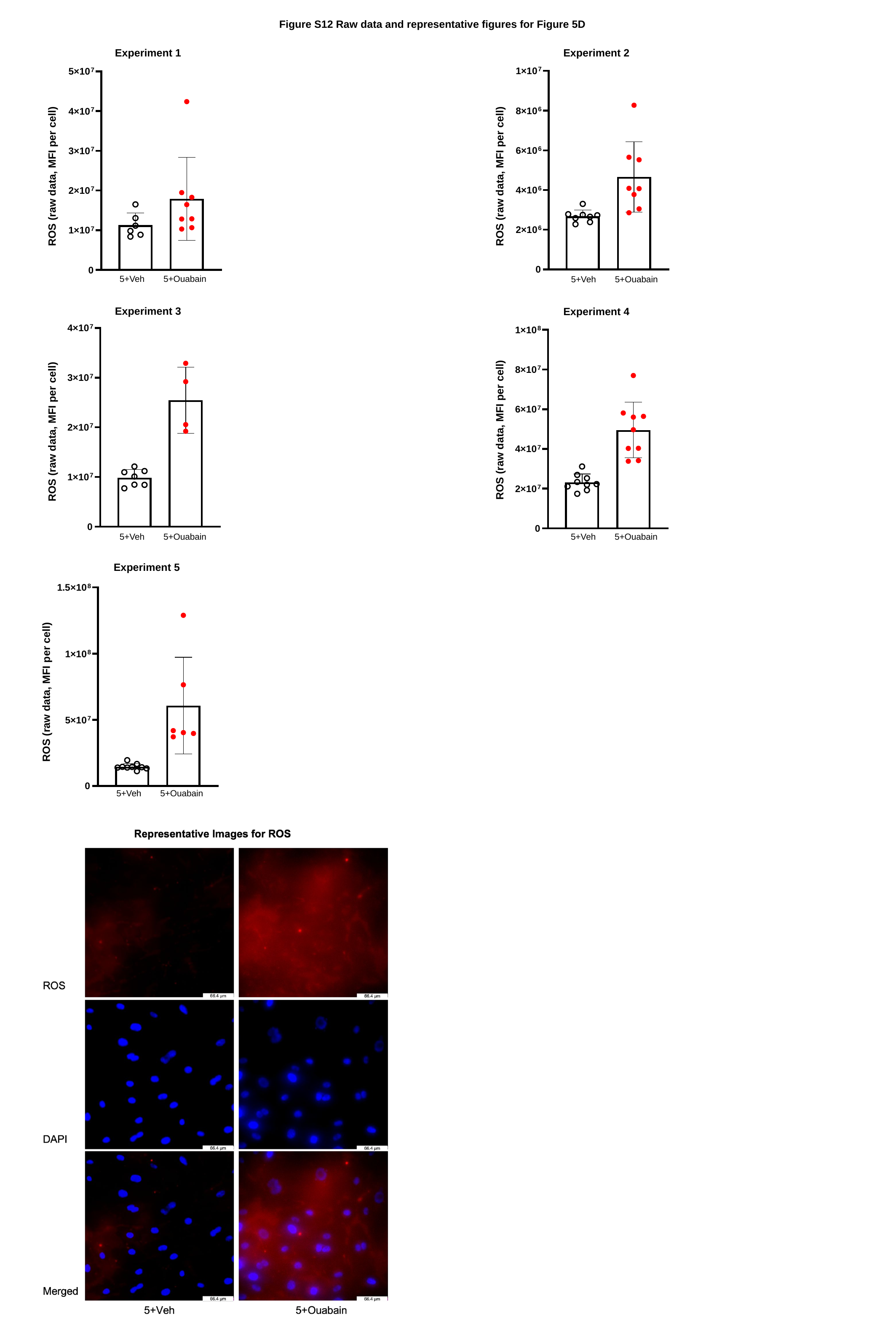

Figure S12 Raw data and representative figures for Figure 5D
Experiment 1
Experiment 2
ROS (raw data, MFI per cell)
ROS (raw data, MFI per cell)
5+Veh
5+Ouabain
5+Veh
5+Ouabain
Experiment 3
Experiment 4
ROS (raw data, MFI per cell)
ROS (raw data, MFI per cell)
5+Veh
5+Ouabain
5+Veh
5+Ouabain
Experiment 5
ROS (raw data, MFI per cell)
5+Veh
5+Ouabain

## Slide 14
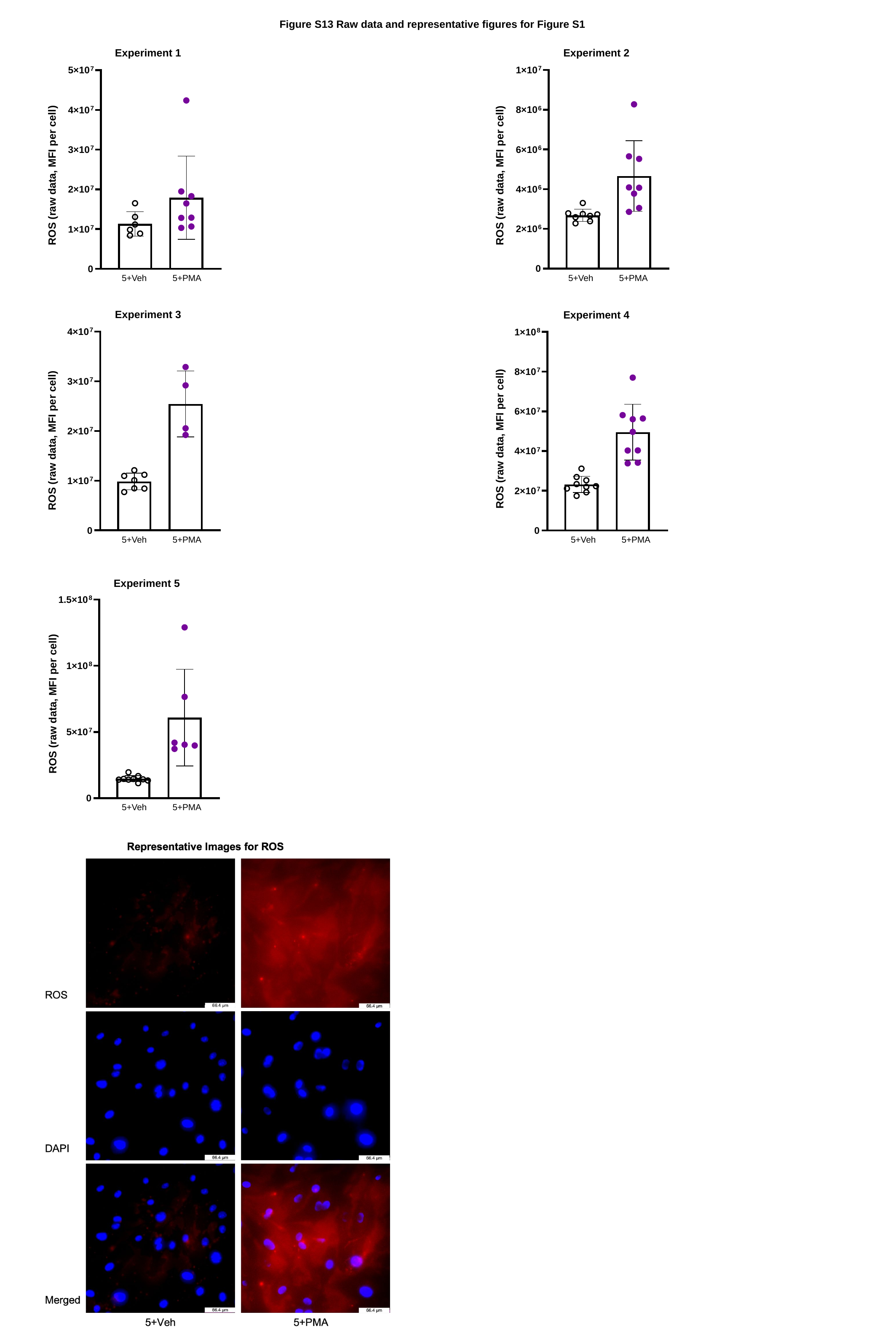

Figure S13 Raw data and representative figures for Figure S1
Experiment 1
Experiment 2
ROS (raw data, MFI per cell)
ROS (raw data, MFI per cell)
5+Veh
5+PMA
5+Veh
5+PMA
Experiment 3
Experiment 4
ROS (raw data, MFI per cell)
ROS (raw data, MFI per cell)
5+Veh
5+PMA
5+Veh
5+PMA
Experiment 5
ROS (raw data, MFI per cell)
5+Veh
5+PMA

## Slide 15
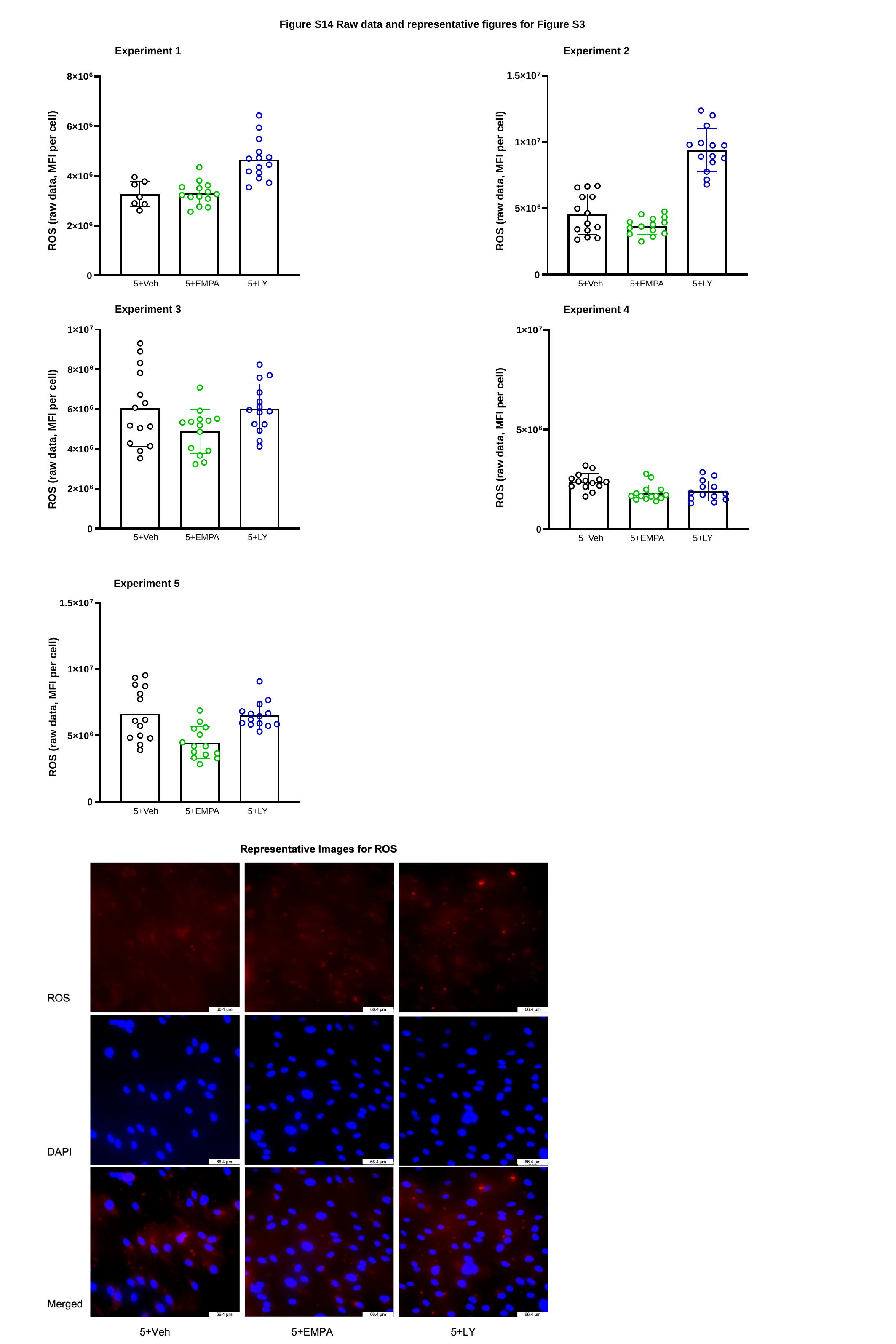

Figure S14 Raw data and representative figures for Figure S3
Experiment 1
Experiment 2
ROS (raw data, MFI per cell)
ROS (raw data, MFI per cell)
5+Veh
5+EMPA 5+LY
5+Veh
5+EMPA 5+LY
Experiment 3
Experiment 4
ROS (raw data, MFI per cell)
ROS (raw data, MFI per cell)
5+Veh
5+EMPA 5+LY
5+Veh
5+EMPA 5+LY
Experiment 5
ROS (raw data, MFI per cell)
5+Veh
5+EMPA 5+LY

## Slide 16
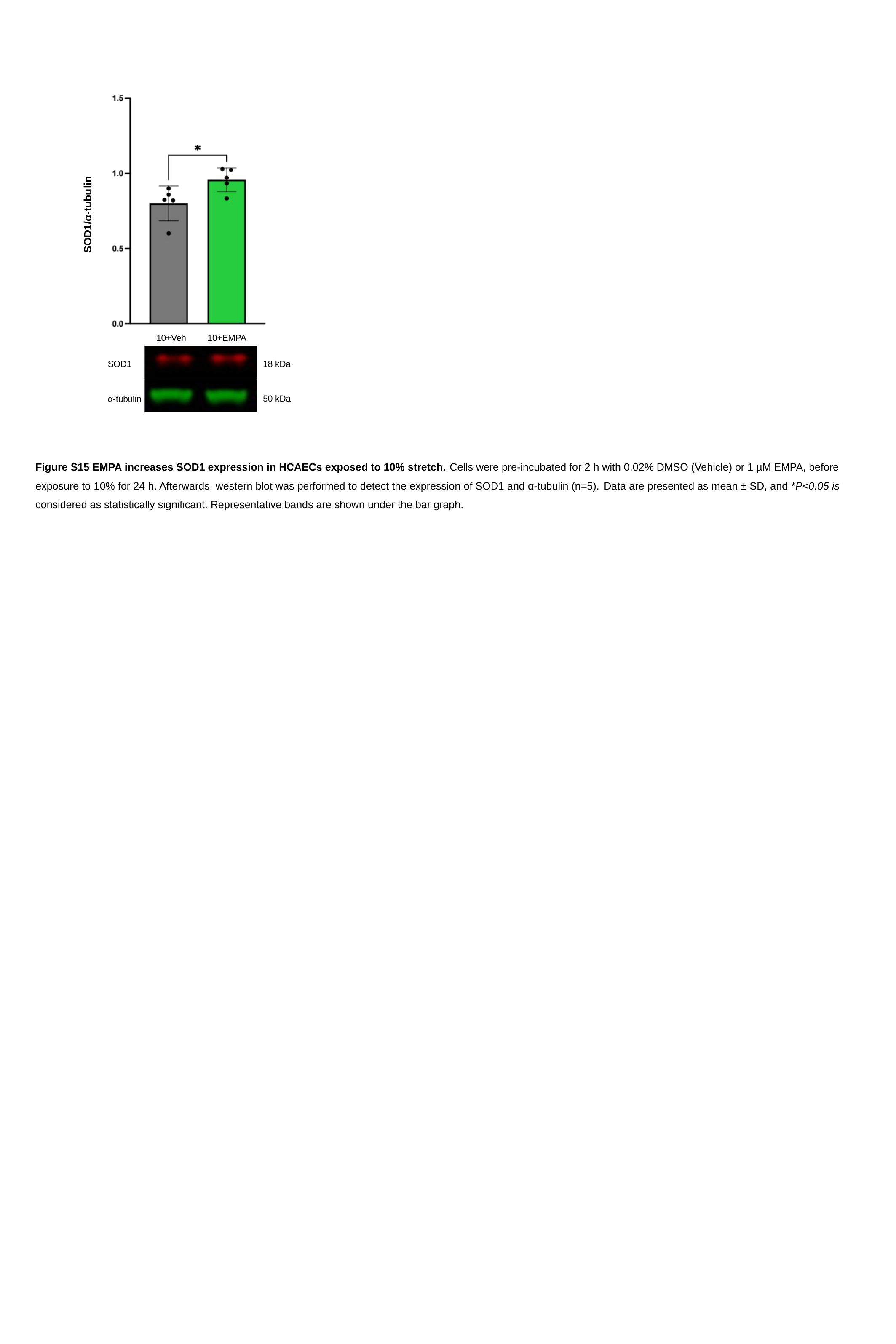

SOD1/α-tubulin
10+Veh
10+EMPA
SOD1
18 kDa
50 kDa
α-tubulin
Figure S15 EMPA increases SOD1 expression in HCAECs exposed to 10% stretch. Cells were pre-incubated for 2 h with 0.02% DMSO (Vehicle) or 1 µM EMPA, before exposure to 10% for 24 h. Afterwards, western blot was performed to detect the expression of SOD1 and α-tubulin (n=5). Data are presented as mean ± SD, and *P<0.05 is considered as statistically significant. Representative bands are shown under the bar graph.

## Slide 17
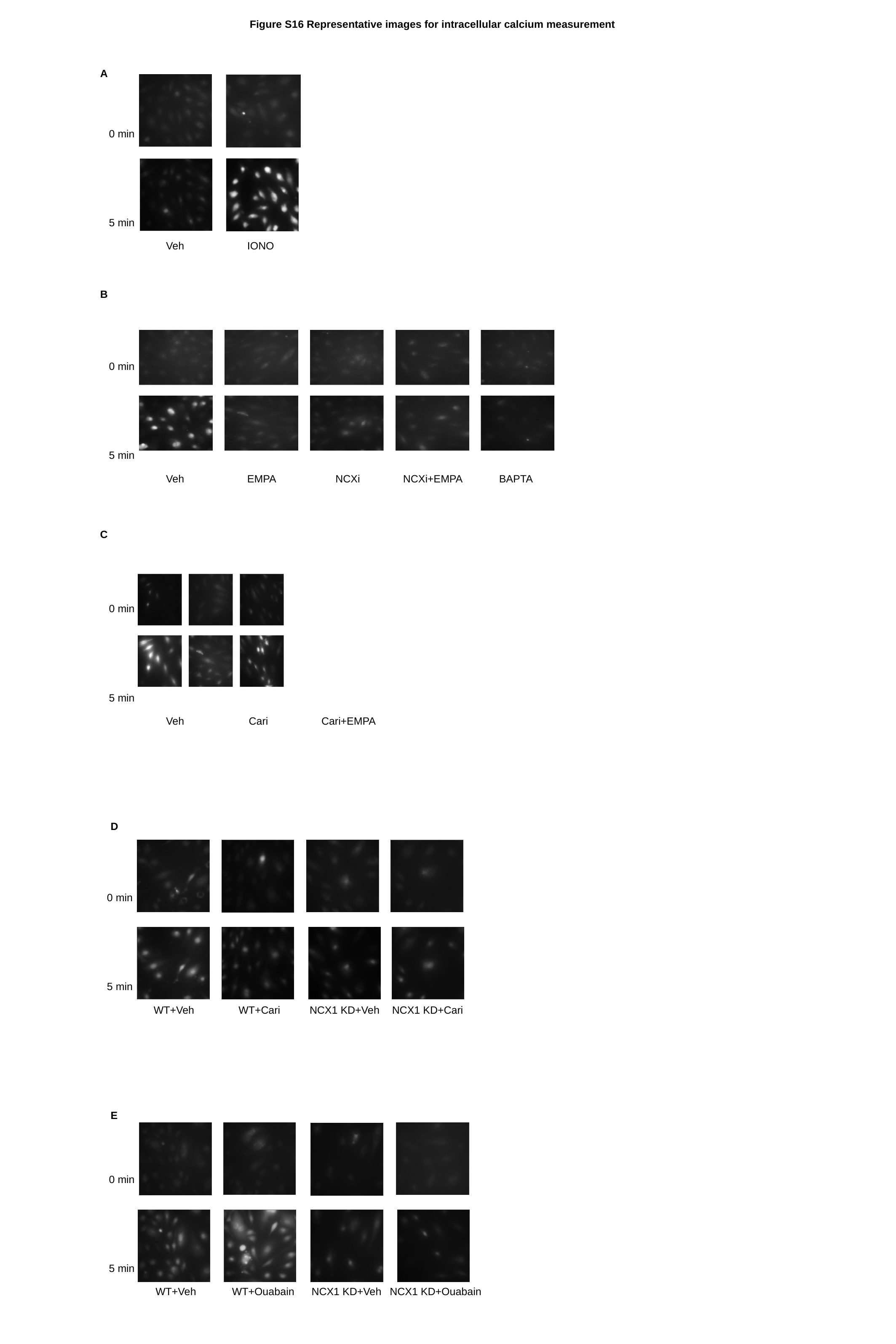

Figure S16 Representative images for intracellular calcium measurement
A
0 min
5 min
Veh
IONO
B
Veh
EMPA
NCXi
NCXi+EMPA
BAPTA
0 min
5 min
C
Veh
Cari
Cari+EMPA
0 min
5 min
D
0 min
5 min
WT+Veh
WT+Cari
NCX1 KD+Veh
NCX1 KD+Cari
E
0 min
5 min
WT+Veh
WT+Ouabain
NCX1 KD+Veh
NCX1 KD+Ouabain

## Slide 18
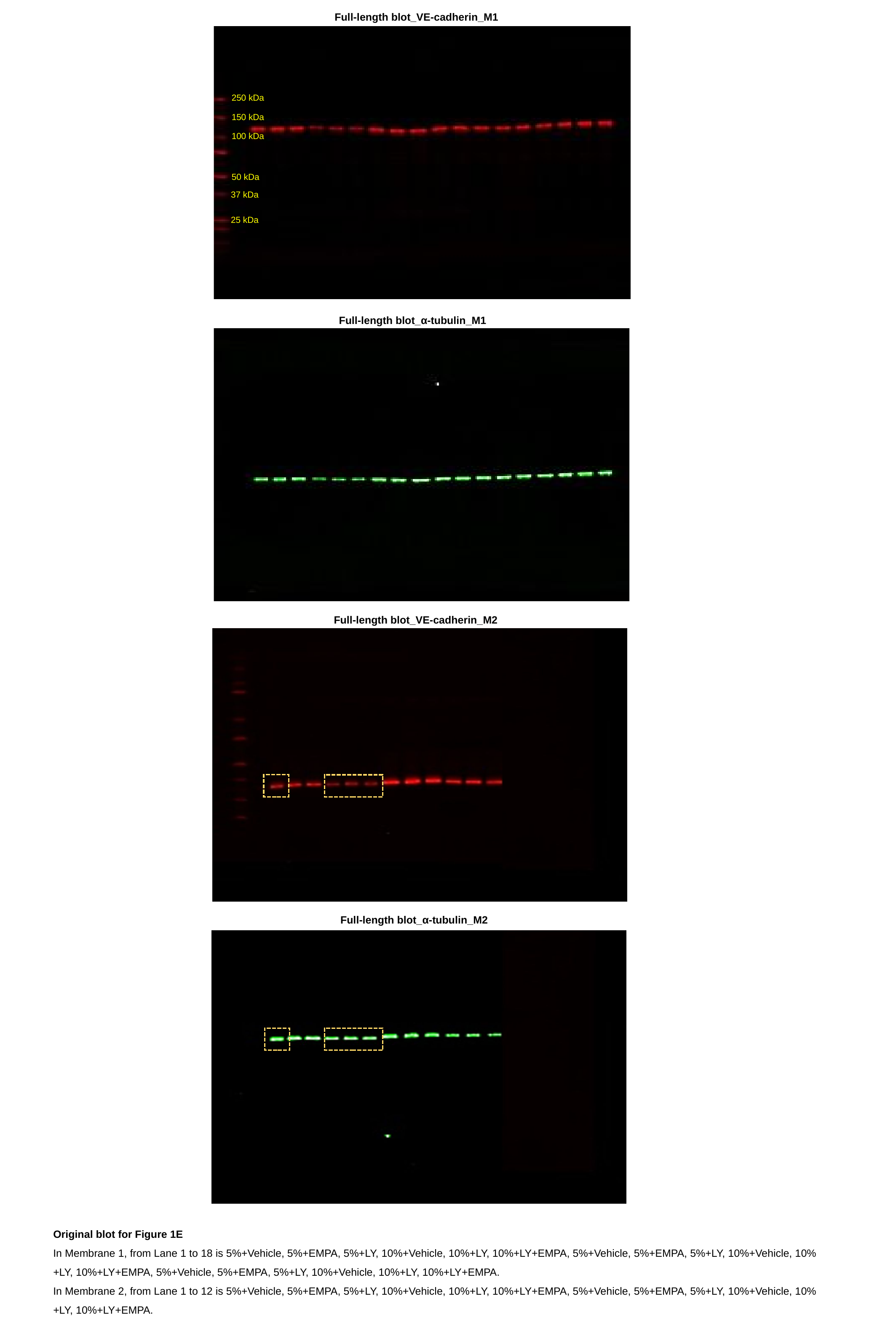

Full-length blot_VE-cadherin_M1
250 kDa
150 kDa
100 kDa
50 kDa
37 kDa
25 kDa
Full-length blot_α-tubulin_M1
Full-length blot_VE-cadherin_M2
Full-length blot_α-tubulin_M2
Original blot for Figure 1E
In Membrane 1, from Lane 1 to 18 is 5%+Vehicle, 5%+EMPA, 5%+LY, 10%+Vehicle, 10%+LY, 10%+LY+EMPA, 5%+Vehicle, 5%+EMPA, 5%+LY, 10%+Vehicle, 10%+LY, 10%+LY+EMPA, 5%+Vehicle, 5%+EMPA, 5%+LY, 10%+Vehicle, 10%+LY, 10%+LY+EMPA.
In Membrane 2, from Lane 1 to 12 is 5%+Vehicle, 5%+EMPA, 5%+LY, 10%+Vehicle, 10%+LY, 10%+LY+EMPA, 5%+Vehicle, 5%+EMPA, 5%+LY, 10%+Vehicle, 10%+LY, 10%+LY+EMPA.

## Slide 19
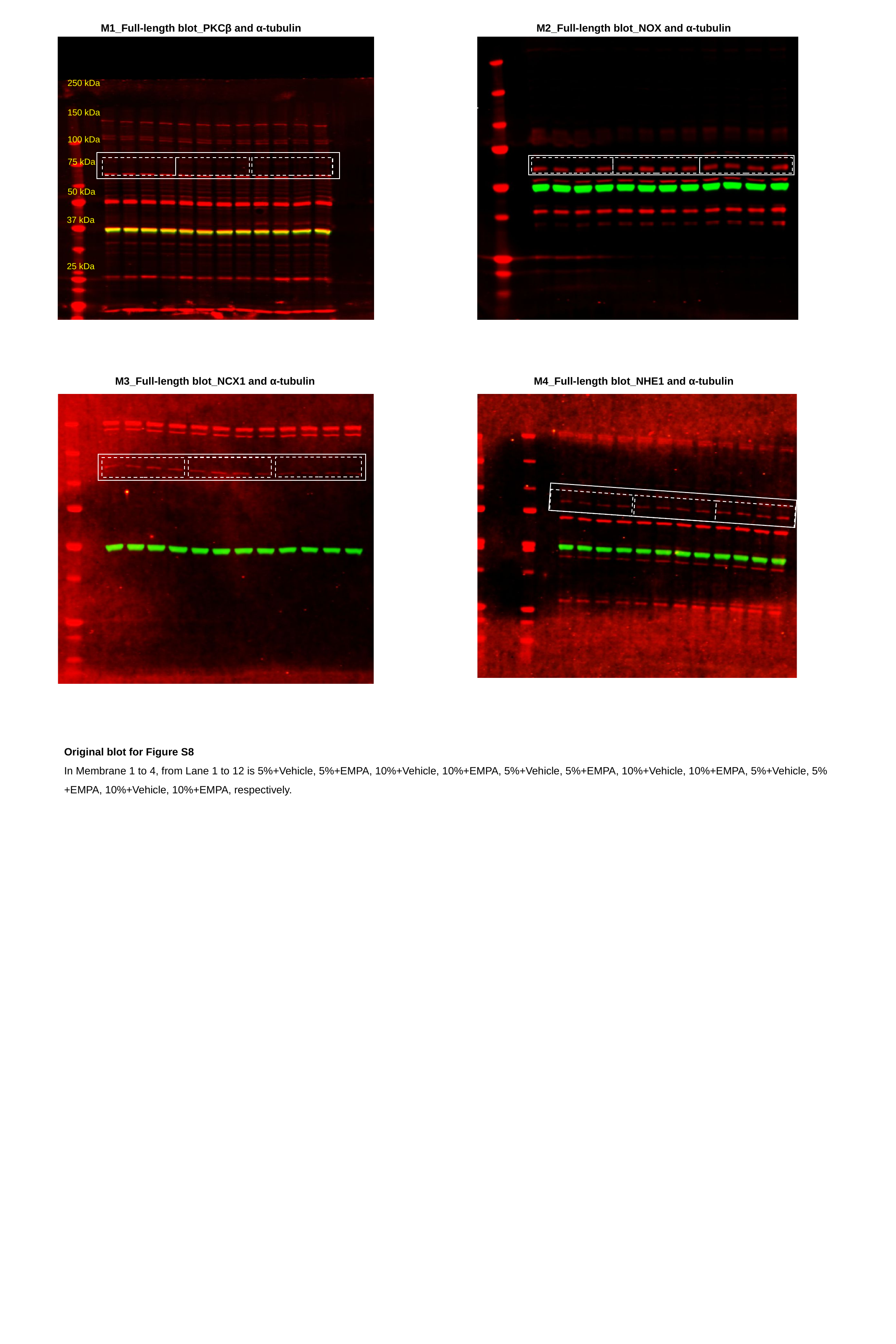

M1_Full-length blot_PKCβ and α-tubulin
M2_Full-length blot_NOX and α-tubulin
250 kDa
150 kDa
100 kDa
75 kDa
50 kDa
37 kDa
25 kDa
M3_Full-length blot_NCX1 and α-tubulin
M4_Full-length blot_NHE1 and α-tubulin
Original blot for Figure S8
In Membrane 1 to 4, from Lane 1 to 12 is 5%+Vehicle, 5%+EMPA, 10%+Vehicle, 10%+EMPA, 5%+Vehicle, 5%+EMPA, 10%+Vehicle, 10%+EMPA, 5%+Vehicle, 5%+EMPA, 10%+Vehicle, 10%+EMPA, respectively.

## Slide 20
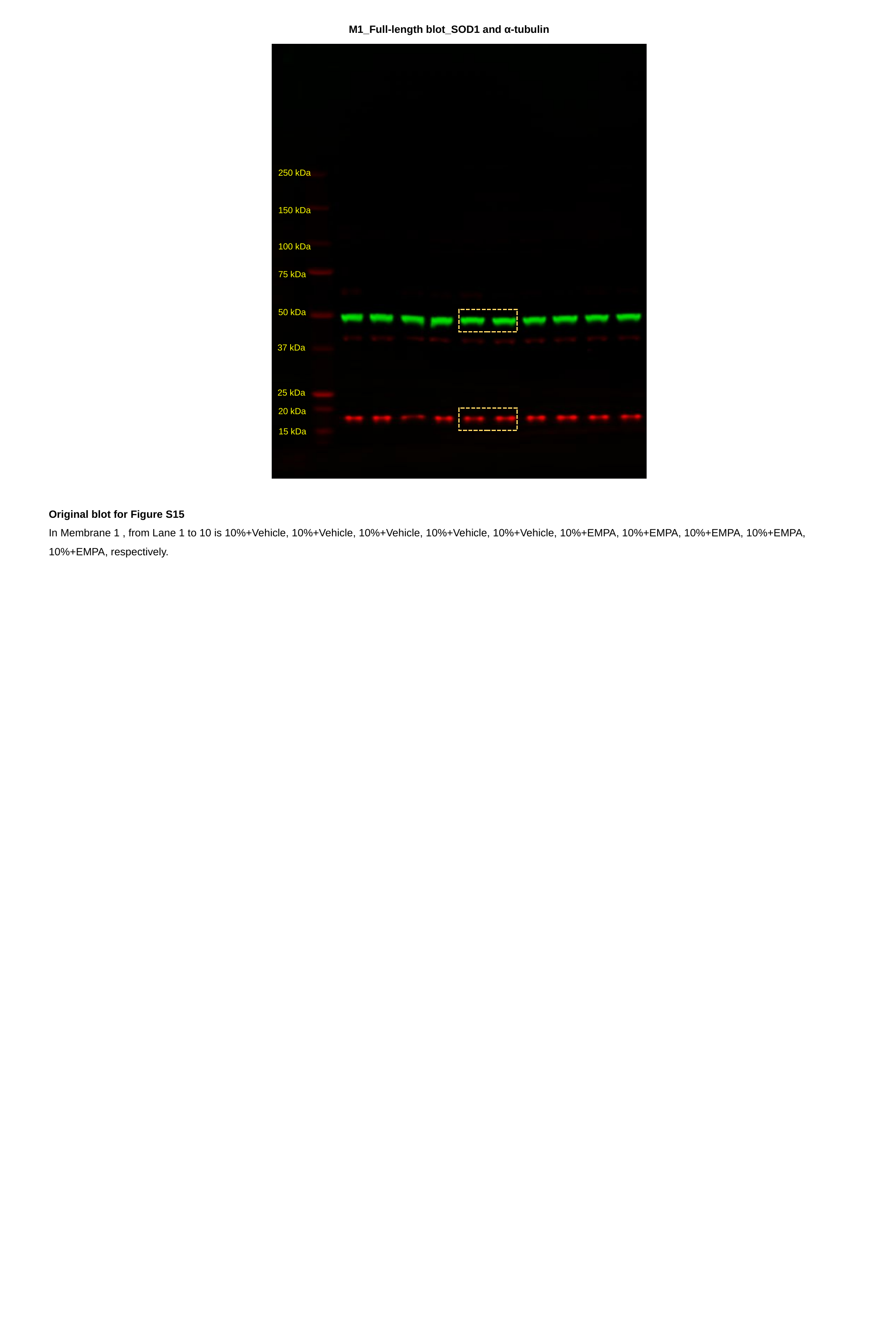

M1_Full-length blot_SOD1 and α-tubulin
250 kDa
150 kDa
100 kDa
75 kDa
50 kDa
37 kDa
25 kDa
20 kDa
15 kDa
Original blot for Figure S15
In Membrane 1 , from Lane 1 to 10 is 10%+Vehicle, 10%+Vehicle, 10%+Vehicle, 10%+Vehicle, 10%+Vehicle, 10%+EMPA, 10%+EMPA, 10%+EMPA, 10%+EMPA, 10%+EMPA, respectively.

## Slide 21
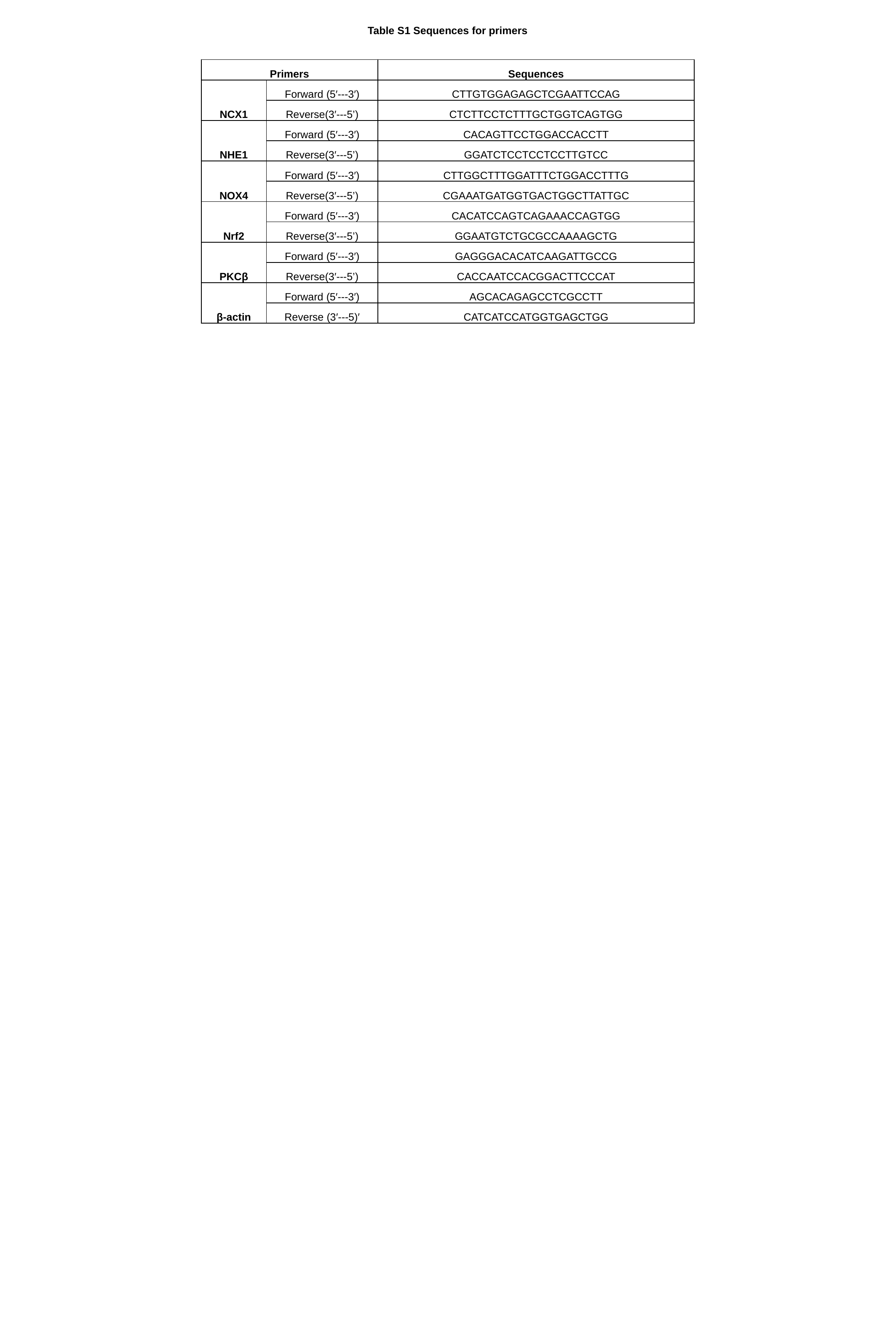

Table S1 Sequences for primers
| Primers | | Sequences |
| --- | --- | --- |
| NCX1 | Forward (5′---3′) | CTTGTGGAGAGCTCGAATTCCAG |
| | Reverse(3′---5’) | CTCTTCCTCTTTGCTGGTCAGTGG |
| NHE1 | Forward (5′---3′) | CACAGTTCCTGGACCACCTT |
| | Reverse(3′---5’) | GGATCTCCTCCTCCTTGTCC |
| NOX4 | Forward (5′---3′) | CTTGGCTTTGGATTTCTGGACCTTTG |
| | Reverse(3′---5’) | CGAAATGATGGTGACTGGCTTATTGC |
| Nrf2 | Forward (5′---3′) | CACATCCAGTCAGAAACCAGTGG |
| | Reverse(3′---5’) | GGAATGTCTGCGCCAAAAGCTG |
| PKCβ | Forward (5′---3′) | GAGGGACACATCAAGATTGCCG |
| | Reverse(3′---5’) | CACCAATCCACGGACTTCCCAT |
| β-actin | Forward (5′---3′) | AGCACAGAGCCTCGCCTT |
| | Reverse (3′---5)′ | CATCATCCATGGTGAGCTGG |

## Slide 22
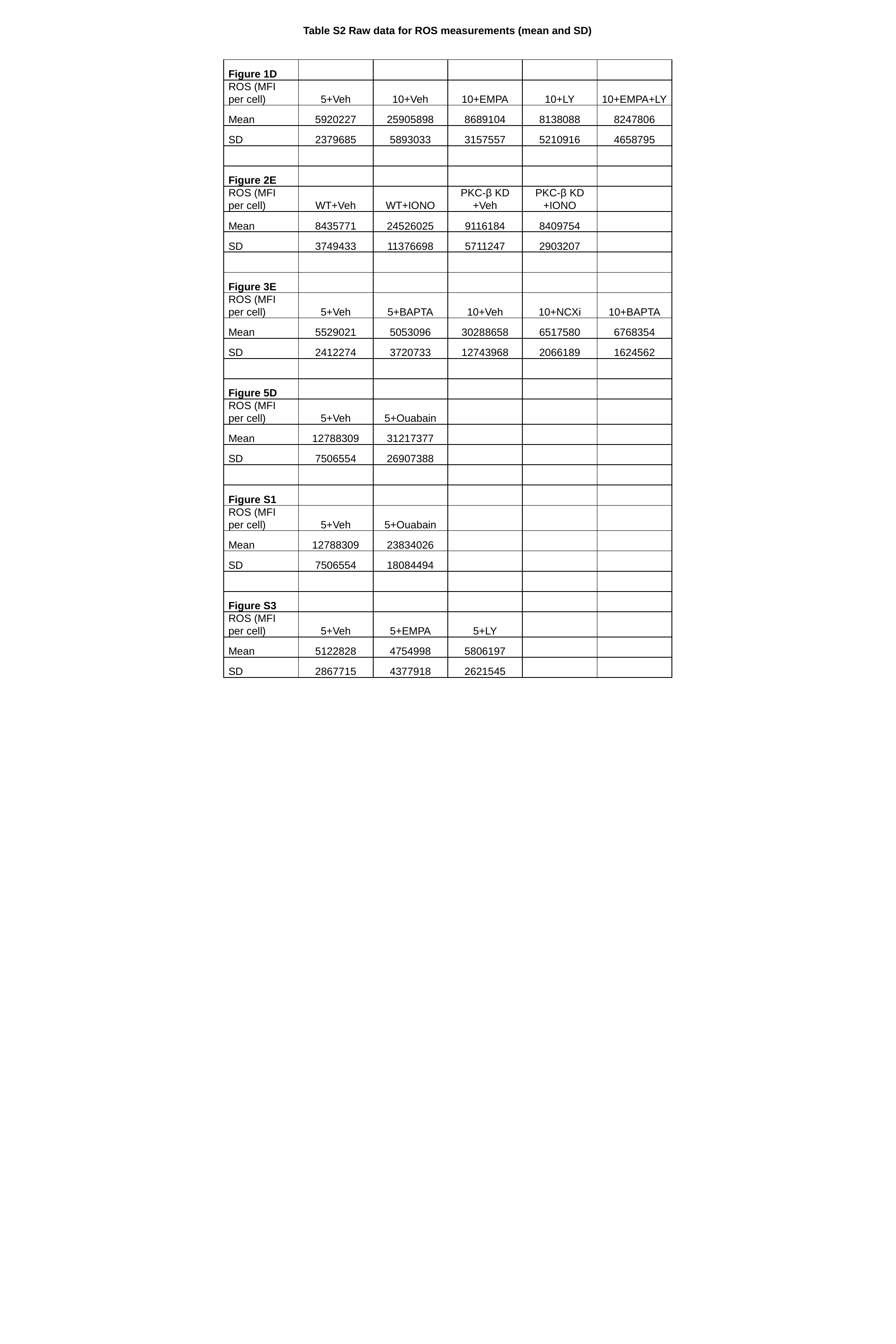

Table S2 Raw data for ROS measurements (mean and SD)
| Figure 1D | | | | | |
| --- | --- | --- | --- | --- | --- |
| ROS (MFI per cell) | 5+Veh | 10+Veh | 10+EMPA | 10+LY | 10+EMPA+LY |
| Mean | 5920227 | 25905898 | 8689104 | 8138088 | 8247806 |
| SD | 2379685 | 5893033 | 3157557 | 5210916 | 4658795 |
| | | | | | |
| Figure 2E | | | | | |
| ROS (MFI per cell) | WT+Veh | WT+IONO | PKC-β KD +Veh | PKC-β KD +IONO | |
| Mean | 8435771 | 24526025 | 9116184 | 8409754 | |
| SD | 3749433 | 11376698 | 5711247 | 2903207 | |
| | | | | | |
| Figure 3E | | | | | |
| ROS (MFI per cell) | 5+Veh | 5+BAPTA | 10+Veh | 10+NCXi | 10+BAPTA |
| Mean | 5529021 | 5053096 | 30288658 | 6517580 | 6768354 |
| SD | 2412274 | 3720733 | 12743968 | 2066189 | 1624562 |
| | | | | | |
| Figure 5D | | | | | |
| ROS (MFI per cell) | 5+Veh | 5+Ouabain | | | |
| Mean | 12788309 | 31217377 | | | |
| SD | 7506554 | 26907388 | | | |
| | | | | | |
| Figure S1 | | | | | |
| ROS (MFI per cell) | 5+Veh | 5+Ouabain | | | |
| Mean | 12788309 | 23834026 | | | |
| SD | 7506554 | 18084494 | | | |
| | | | | | |
| Figure S3 | | | | | |
| ROS (MFI per cell) | 5+Veh | 5+EMPA | 5+LY | | |
| Mean | 5122828 | 4754998 | 5806197 | | |
| SD | 2867715 | 4377918 | 2621545 | | |
